# Molecular Mechanisms in the Activation of Abscisic Acid Receptor PYR1

**DOI:** 10.1371/journal.pcbi.1003114

**Published:** 2013-06-27

**Authors:** Lyudmyla Dorosh, Olesya A. Kharenko, Nandhakishore Rajagopalan, Michele C. Loewen, Maria Stepanova

**Affiliations:** 1 National Research Council of Canada, Edmonton, Alberta, Canada; 2 Department of Electrical and Computer Engineering, University of Alberta, Edmonton, Alberta, Canada; 3 National Research Council of Canada, Saskatoon, Saskatchewan, Canada; 4 Department of Biochemistry, University of Saskatchewan, Saskatoon, Saskatchewan, Canada; UNC Charlotte, United States of America

## Abstract

The pyrabactin resistance 1 (PYR1)/PYR1-like (PYL)/regulatory component of abscisic acid (ABA) response (RCAR) proteins comprise a well characterized family of ABA receptors. Recent investigations have revealed two subsets of these receptors that, in the absence of ABA, either form inactive homodimers (PYR1 and PYLs 1–3) or mediate basal inhibition of downstream target type 2C protein phosphatases (PP2Cs; PYLs 4–10) respectively *in vitro*. Addition of ABA has been shown to release the apo-homodimers yielding ABA-bound monomeric holo-receptors that can interact with PP2Cs; highlighting a competitive-interaction process. Interaction selectivity has been shown to be mediated by subtle structural variations of primary sequence and ligand binding effects. Now, the dynamical contributions of ligand binding on interaction selectivity are investigated through extensive molecular dynamics (MD) simulations of apo and holo-PYR1 in monomeric and dimeric form as well as in complex with a PP2C, homology to ABA insensitive 1 (HAB1). Robust comparative interpretations were enabled by a novel essential collective dynamics approach. In agreement with recent experimental findings, our analysis indicates that ABA-bound PYR1 should efficiently bind to HAB1. However, both ABA-bound and ABA-extracted PYR1-HAB1 constructs have demonstrated notable similarities in their dynamics, suggesting that apo-PYR1 should also be able to make a substantial interaction with PP2Cs, albeit likely with slower complex formation kinetics. Further analysis indicates that both ABA-bound and ABA-free PYR1 in complex with HAB1 exhibit a higher intra-molecular structural stability and stronger inter-molecular dynamic correlations, in comparison with either holo- or apo-PYR1 dimers, supporting a model that includes apo-PYR1 in complex with HAB1. This possibility of a conditional functional apo-PYR1-PP2C complex was validated *in vitro*. These findings are generally consistent with the competitive-interaction model for PYR1 but highlight dynamical contributions of the PYR1 structure in mediating interaction selectivity suggesting added degrees of complexity in the regulation of the competitive-inhibition.

## Author Summary

Protein pyrabactin resistance 1 (PYR1) belongs to a group of PYR1-like (PYL) proteins that regulate plant development and responses to conditions of drought and salinity. Recent studies have reported characterization of their molecular structures as well as elucidation of important aspects of their function; highlighting their roles as receptors for the stress responsive phytohormone, abscisic acid (ABA). However details of the molecular mechanisms regulating their receptor signalling remain enigmatic. In this work, we use molecular dynamics simulations complemented by a sophisticated statistical-mechanical analysis to investigate structural and dynamical properties of PYR1 protein and how its interaction with ABA modifies receptor-protein complex formation. Our results provide detailed insight into how the PYR1-mediated inactivation of its downstream phosphatase target is regulated by homodimer formation and yield new hypotheses, supported by *in vitro* experiments, for further investigation. Ultimately, this knowledge provides insight into how plants respond to stress, with potential applications in the development of crops with improved growth characteristics and higher stress tolerance.

## Introduction

The plant hormone abscisic acid (ABA) controls seed development, germination, dormancy and stress response [1], [2]. Using combined molecular, biophysical and genetic techniques many details of ABA signal transduction have been elucidated [3]–[5]. In particular, under abiotic stress, such as drought and high salinity, ABA levels have been shown to increase in the plant, initiating adaptive responses involving inhibition of type 2C protein phosphatases (PP2C), and stimulation of protein sucrose non-fermenting related kinases 2 (SnRK2). In 2009, two research groups independently reported a family of at least 13 ABA-binding proteins in *Arabidopsis thaliana*, known as PYR1 (pyrabactin resistance 1) and PYL (PYR1-like) or RCAR (regulatory component of ABA response) proteins, that play a central role in ABA signal transduction mediating PP2C inhibition [6]–[8].

**Figure 1:**
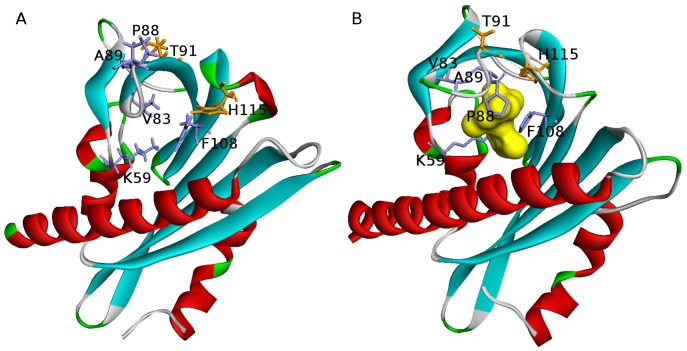
Abscisic acid binding by the PYR1 ligand pocket induces gate-latch-locking. (A) – Structure of the apo-PYR1, gate open [PDB ID 3K3K, chain A]. (B) – Structure of ABA-bound PYR1, gate closed [PDB ID 3K3K, chain B]. The lock mechanism involves both direct and water/ions-mediated interactions of residues from gate (residues 85–89) and latch (residues 115–117), as well as hydrophobic interactions and hydrogen bonds throughout the binding pocket's surface. Residues which contribute to hydrogen binding in gate and latch are labeled and shown by orange sticks, while hydrophobic residues in the neighborhood of ABA (colored yellow) are shown by purple sticks. The allosteric rearrangement of gate and latch loops forms a surface for successful PP2C binding. Upon the binding, a conserved PP2C tryptophan 385 (not shown) is inserted between gate and latch and forms water-mediated hydrogen bond with ABA [9], [11].

The structure of a typical PYR/PYL receptor comprises seven β-strands and two α-helices [9]–[11]. The second α-helix, located in the C-terminal region of PYR1, forms a helix-grip fold, which in turn provides a large hydrophobic cavity, the ligand pocket (Figure 1). Upon binding to the receptor, ABA makes direct stabilizing contacts with two flexible loops (Lβ3β4 and Lβ5β6 named gate/proline cap and latch/leucine lock), which then act as a scaffold mediating an interaction with the PP2C [11]. In the course of PYR/PYL receptors docking to PP2Cs, some side chains of the gate (e.g. conserved S85 of PYR1) interact with the phosphatase in such a way as to block access to the PP2C catalytic site [12], [11]. The overall effect of PYR/PYL binding to PP2C is inhibition of the phosphatase which can then no longer inhibit SnRK2 activity. As a result, active SnRK2 phosphorylates and activates downstream transcription factors leading to well documented ABA-responsive gene induction events [13].

More recently it has been found that PYR1 and PYLs 1–3 generally do not show basal activity, whereas other family members PYLs 4–10 (not including untested PYL7) are constitutively active (CA) *in vitro* [7], [14], [15]. Interestingly however, PYL4 was only active against HAB1 under the conditions tested [15]. Indeed, the demonstrated ability of PYL10 to strongly inhibit PP2C even in the absence of any ligand has been employed to engineer a gate-modified mutant of PYL2 (V87L) with improved basal activity [15]. Mechanistically, the ability of a native receptor to mediate basal activity has been correlated to its inability to homodimerize in solution [15], [16]. According to published structural data, the receptor's binding surfaces involved in the formation of dimers and complexes with PP2C mainly overlap [8], [10], [17] suggesting a possibility of competition between dimerization and PP2C docking. In this context, it has been shown that mutation I88K in PYL2 both prevents dimer formation in solution and increases its constitutive activity [15]. PYR1 mutation H60P was also found to yield a mixture of monomeric and dimeric PYR1 forms, which showed weak basal activity [16], [18]. While these mutations may explain why PYLs 8–10 tend to be monomeric, factors contributing to PYLs 4–6 being monomeric remain unknown. Together these reports show that for homodimeric receptors such as wild type PYR1, ABA binding prevents dimerization, induces dimer dissociation and stimulates receptor-PP2C interactions.

Toward characterizing molecular aspects regulating basal versus inducible receptor activity against PP2Cs, numerical modeling and simulations are required. Molecular dynamics (MD) simulations have been reported involving phosphatase proteins other than PP2C. For example, a system comprising the N-terminal part of phosphatase SHP-2 and a peptide (101 residues) has been studied using MD simulations for 10 ns [19], and a somewhat larger complex of protein-tyrosine phosphatase 1B (337 residues) with phosphorylated peptide substrate (193 residues) [20] has been simulated for 1 ns. At the time of writing, the only modeling work on PYR1 and PYL1 [21] is addressing the design of small ligands that could replace ABA, improving the ligand binding energy. However in order to elucidate mechanistic information about the receptors more broadly, the entire PYR/PYL-PP2C complex needs to be simulated. The complexity is that MD simulations of such a system, which consists of approximately 8,000 atoms, or 530 residues, embedded in an explicit water solvent of approximately 60,000 atoms, at physiologically relevant timescales (≥100 ns) are prohibitively computationally expensive. Toward overcoming this problem, various dimensionality reduction or coarse grained approaches may be employed to draw predictions from available MD simulations [22]–[29]. In these approaches, collective dynamical descriptors are derived from MD data by such techniques as the principal component analysis (PCA) [23], [24], normal mode analysis [30], [27], or related methods, expecting that relevant structural properties, such as the peptide flexibility, could be extracted [28], [25], [20].

Toward enabling robust interpretations, a novel essential collective dynamics (ECD) modeling framework has been recently introduced, which allows probing of persistent dynamic correlations in proteins based on short (a few hundreds of picoseconds) all-atom MD trajectories [31]–[35]. Relying on a statistical-mechanical theory, the ECD framework provides a transparent physical interpretation of the dimensionality reduction analyses in terms of a proteins' structural properties such as the main chain flexibility or dynamic domains of correlated motion, as well as allows for a reasonable match of the corresponding predictions with NMR-based measurements representing significantly longer time regimes than the MD trajectories used in the analysis.

In this work, extensive MD simulations for PYR1 bound to HAB1 phosphatase as well as PYR1 homodimer [10], [17] are reported in the presence and absence of ligand. Employing the ECD framework the structural stability and dynamics of PYR1 complexes were investigated Employing the ECD framework the structural stability and dynamics of PYR1 complexes were investigated. The results are consistent with the ABA-dependent ‘competitive interaction’ model (receptor homodimer versus receptor-phosphatase complexes [16]) proposed for regulation of PYR1, suggest a stronger potential role for ABA-independent, concentration-dependent regulation of basal signaling for all PYR1/PYL receptors and define the dynamical contributions that mediate the selective interactions of PYR1.

## Results/Discussion

### Optimization of PYR1 Simulations and ECD Analyses

Molecular models of PYR1 monomers and dimers as well as PYR1-PP2C complexes in water were constructed employing crystallographic models from the Protein Data Bank (PDB) [36], see also Table 1 and Methods section. Molecular dynamics simulations were carried out for these constructs and the results analyzed by the ECD method [31]–[34] as described in Methods.

**Table 1:** List of 3D PYR1 constructs taken from PDB and modeled *in silico*, which were used for molecular dynamics simulations and the ECD analysis.

Construct	PYR1 monomer	PYR1 dimer	PYR1-HAB1 complex
Ligand free	(1) 3K3K PDB ID chain A [9]; (2) 3K3K PDB ID chain B [9], ABA-extracted	PYV/P2M extracted from 3NJO PDB ID [10], S88 replaced with P88.	(1) ABA extracted from 3QN1 PDB ID [17]; (2) PYR1 shifted against HAB1 by 15 Å
ABA-bound	3K3K.pdb chain B [9]	(1) PYV/P2M replaced with two ABA and S88P mutation in 3NJO PDB ID; (2) PYV replaced with one ABA, P2M removed, and S88P mutation in 3NJO PDB ID	(1) 3QN1 PDB ID [17], with parts of HAB1 reconstructed; (2) PYR1 shifted against HAB1 by 15 Å
Pyrabactin bound	ABA replaced with PYV in chain B of 3K3K	3NJO PDB ID chains A, B [10]	

**Figure 2:**
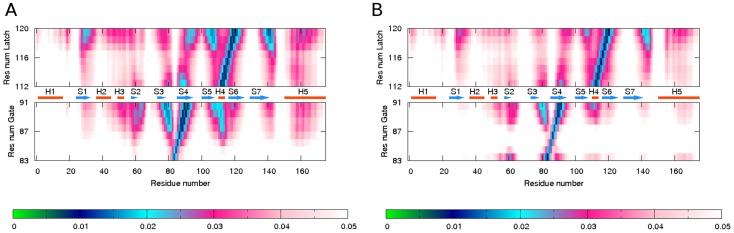
Correlation maps of residues in gate (residues 85–89) and latch (residues 115–117) regions for the PYR1 constructs from Figure 1: (A) – closed lid, ABA-bound, (B) – open lid, ABA-free receptor. The simulations have been performed at 300 K. Strong correlations are represented by low values of the descriptor (green and blue colors), whereas high values indicate a more independent motion (magenta and white colors).

Examples of intra-molecular correlation maps are shown in Figure 2 for the PYR1 open lid and PYR1 closed lid conformations from Figure 1. The maps show ECD pair correlation descriptors 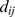 computed by Eq. 1, and represent inter-atomic correlations originating from direct binding, steric constraints, and water-mediated interactions in the constructs considered. From comparison of the correlation maps, a significant loss of correlations is evident for the gate (V83-N90) and latch (E114-T118) regions around residues C30, H60, L87 and M158-L166 in the open lid PYR1 construct. The correlations of the gate with loop Lβ7α5 and helix α5 which are observed in the closed lid conformation are not pronounced in the open lid construct.

**Figure 3:**
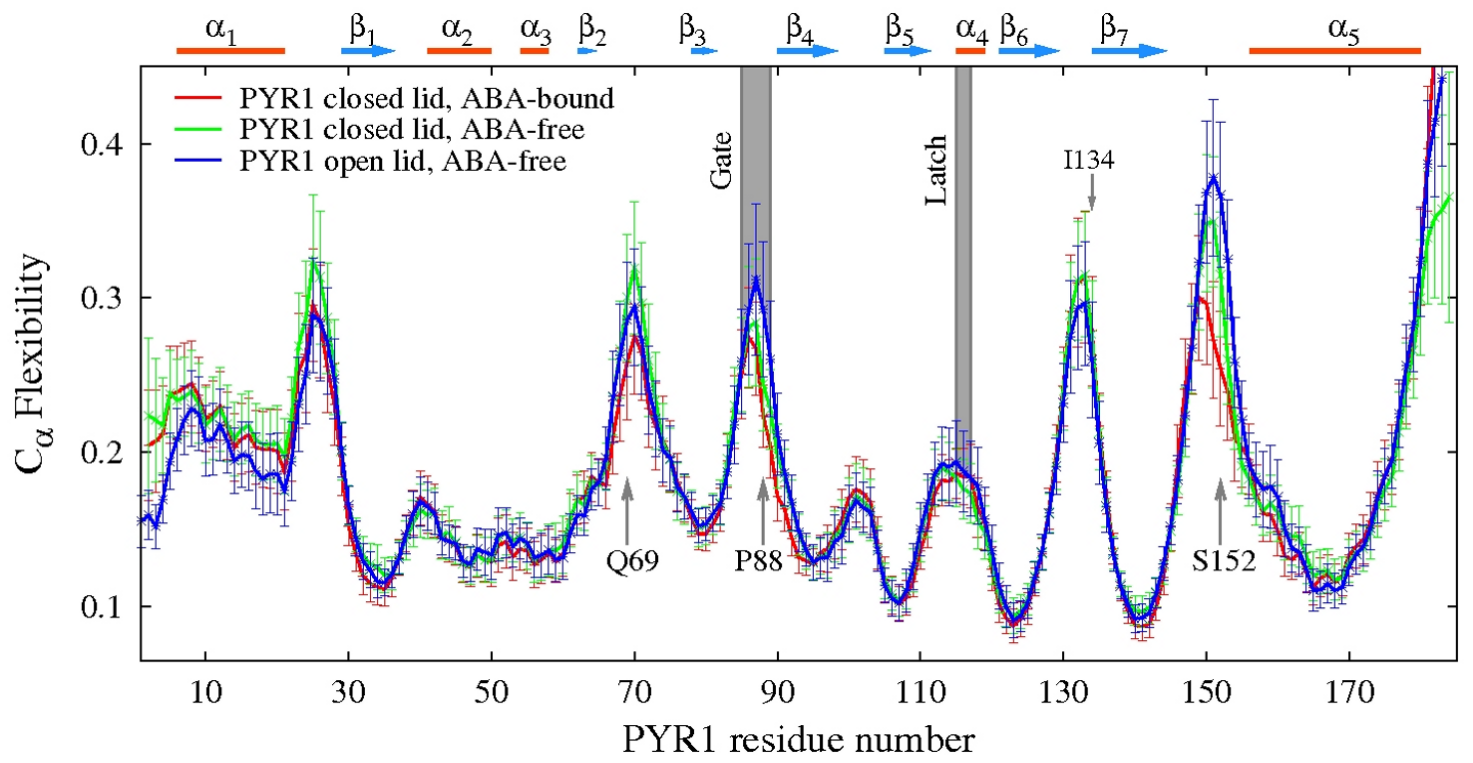
Main-chain flexibility profiles of PYR1-ABA-bound closed lid (red line), PYR1 ABA-free closed lid (green line) and PYR1 ABA-free open lid (blue line) monomer constructs with standard deviations indicated by vertical lines. The simulations were performed at 300 K.

Examples of the resulting main chain ECD flexibility profiles of the various PYR1 constructs are shown in Figure 3. In the flexibility profiles, high levels of the descriptor usually correspond to flexible loops, whereas most of the flexibility minima indicate α-helices and β-sheets, in accordance with other methods of flexibility assessment [25], [37]. In particular, high flexibilities of the loop Lβ7α5 (residues P148-D155) and the gate region (residues 85–89) as well as around residues Q69, I134 and Y23 are observed. The flexibilities of open and closed lid structures in the areas of these loops differ by up to 62%. Interestingly, the flexibility of loop Lβ7α5, which is only indirectly involved in gate closure, is affected even more strongly than that of the gate upon ABA-binding, whereas the latch flexibility is similar in these constructs, reflecting the “recoil motif” interaction upon ABA-affected PYR1 enclosure [9].

**Figure 4:**
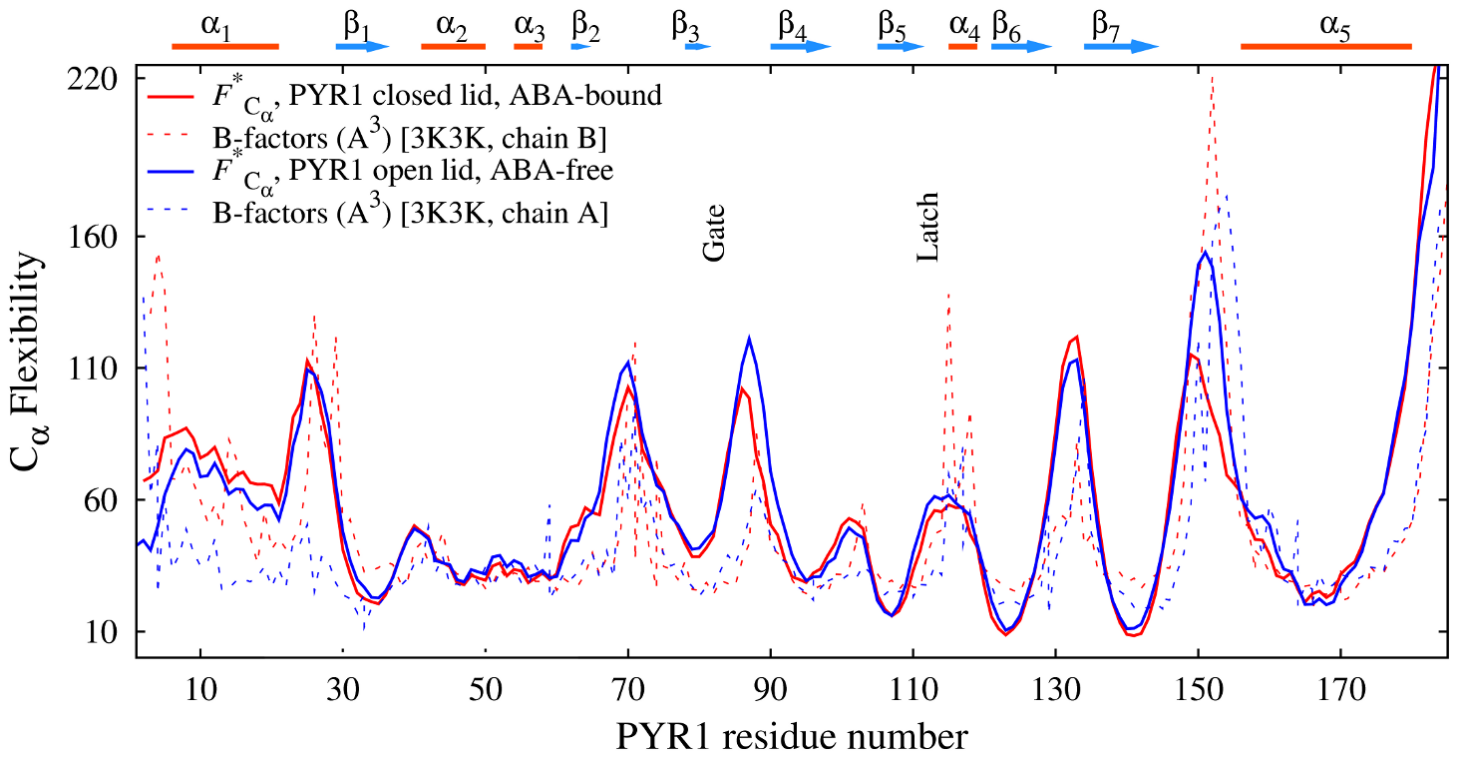
Normalized main-chain flexibility profiles of PYR1 monomers 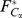
 (solid lines) over-imposed on B-factors of the corresponding starting crystallographic structures (chains A and B from PDB entry 3K3K [9], dashed lines). In the plot, red color represents PYR1 in ABA-bound, closed-lid conformation and blue color represents ABA-free, open lid conformation.

Figure 4 compares our computed flexibility profiles of the PYR1 constructs with crystallographic B factors [9]. B-factors (or Debye-Waller factors) of atoms are derived from X-ray diffraction intensities and are indicative of the relative structural order in the crystal structure. Low crystallographic B-factors correspond to relatively well defined lattice positions, whereas higher B-factors can be interpreted as more flexible and less ordered regions in the crystal structure. While the physical meaning of the crystallographic B factors is somewhat different from the ECD descriptors, and the dynamics of the protein in crystal structure also differs from that in solution, validation of molecular dynamics analyses against B-factors is a popular choice [34]. The backbone B-factors for PYR1 closed lid, ABA-bound construct and PYR1 open lid, ABA-free construct (chains B and A, respectively, from PDB entry 3K3K [9]) are plotted by dashed lines in Figure 4. The computed flexibilities 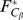 in the figure (solid lines) represent the corresponding profiles from Figure 3, normalized and over-imposed on the experimental profiles to facilitate the comparison. The comparison shows a good agreement in general. For the open-lid construct, the crystallographic B-factors are relatively lower than the ECD flexibility in the N-terminal area and in the gate, whereas for the closed-lid construct, the B-factors are higher than the ECD flexibilities in the latch and in the area of loop Lβ7α5. The reason of the differences around the gate for the open-lid construct is evident and related with constrained motion of the gate in the crystallographic structure. For the closed-lid construct, the relatively high B-factors in the regions of the loops Lβ7α5 and the latch can be attributed to the luck of stabilizing water-mediated hydrogen bonds of loops' residues with the rest of the protein in the phosphatase-binding area of the crystallographic structure. One can conclude that, considering the different physical meaning of B-factors and the ECD flexibilities, the agreement is satisfactory. The differences observed highlight the importance of a thorough analysis of protein dynamics in solution. While the availability of crystallographic structures is major to understand the dynamics, the structures alone do not fully represent important aspects that are addressable by molecular dynamics studies.

**Figure 5:**
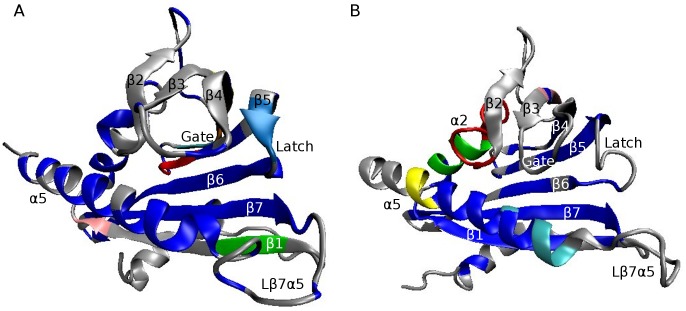
Dynamical domains of correlated motion for the pyrabactin receptor (A) – closed lid, ABA-bound, ABA not shown; (B) – open lid, ABA-free receptor. Simulations were performed at 300 K. Six largest domains are shown, colored blue, red, green, light blue, yellow and pink in the decreasing size order.

Figure 5 presents typical examples of dynamic domains of collective motion in PYR1-ABA-free, open lid construct and PYR1-ABA-bound, closed lid construct obtained by ECD analysis of the MD trajectories as described in Methods. It can be seen that the largest domain in PYR1 ABA-bound construct contains the gate, sheets β7, β6, β5 from the ligand pocket, loops Lβ2β3 and Lβ7α5, and helices α2 and α5. This includes the three main structural motifs of closed lid PYR1 [9] and also the ligand pocket elements. In the open lid construct, the gate and second helix are dynamically uncoupled, and only the central part of helix α5 is correlated with the ligand pocket area.

Finally, simulations for the PYR1 closed lid, ABA-extracted construct were conducted over a range of temperatures including 281 K, 300 K, 310 K and 325 K and analyzed for impact of temperature on dynamics in order to identify a suitable temperature for PYR1 simulations (Figure S1). Interestingly, the closed lid ABA-extracted construct exhibits a stable gate-latch lock, which opened only after a 20 ns simulation at increased temperature (325 K), while the open lid construct showed switching of the gate from closed to open lock positions in the course of all simulations. At 281 K the largest domain of correlated motion, which indicates the most extensive correlations, includes most of the β-sheets, gate and latch, whereas helix α5 is decoupled. At temperatures 300 K and 310 K, the gate and latch are largely decoupled; however most of helix α5 is involved in the largest domain, which contains more than 10% of the receptor's atoms. At 325 K the size of the largest domain decreases and its structure is different from that observed at 300 K and 310 K, indicating as expected, that simulations at 325 K may not be representative of physiologically relevant conditions. Finally, in addition to analyzing the dynamical domains at various temperatures, we also inspected visually the evolution of the ABA-extracted, closed lid PYR1 construct during 50 ns simulations (Figure S2). At 281 K and 300 K temperatures, the conformation remained closed most of the time. In contrast, the contacts of gate and C-terminal helix with the rest of receptor were disrupted after 30 ns of simulations at 310 K and at 325 K. Based on these tests, 310 K has emerged as a condition which allows observation of changes of conformation such as the gate and latch decoupling upon removal of ABA, whereas the dynamics of the receptor are not altered significantly in comparison to 300 K. Therefore 310 K was adopted as a physiologically relevant temperature, yet high enough to allow observation of pertinent conformational evolution *in silico*. The simulations described below are performed at 310 K, unless indicated otherwise.

### ECD Analysis of PYR1-HAB1 Ligand-Free and Ligand-Bound Systems

**Figure 6:**
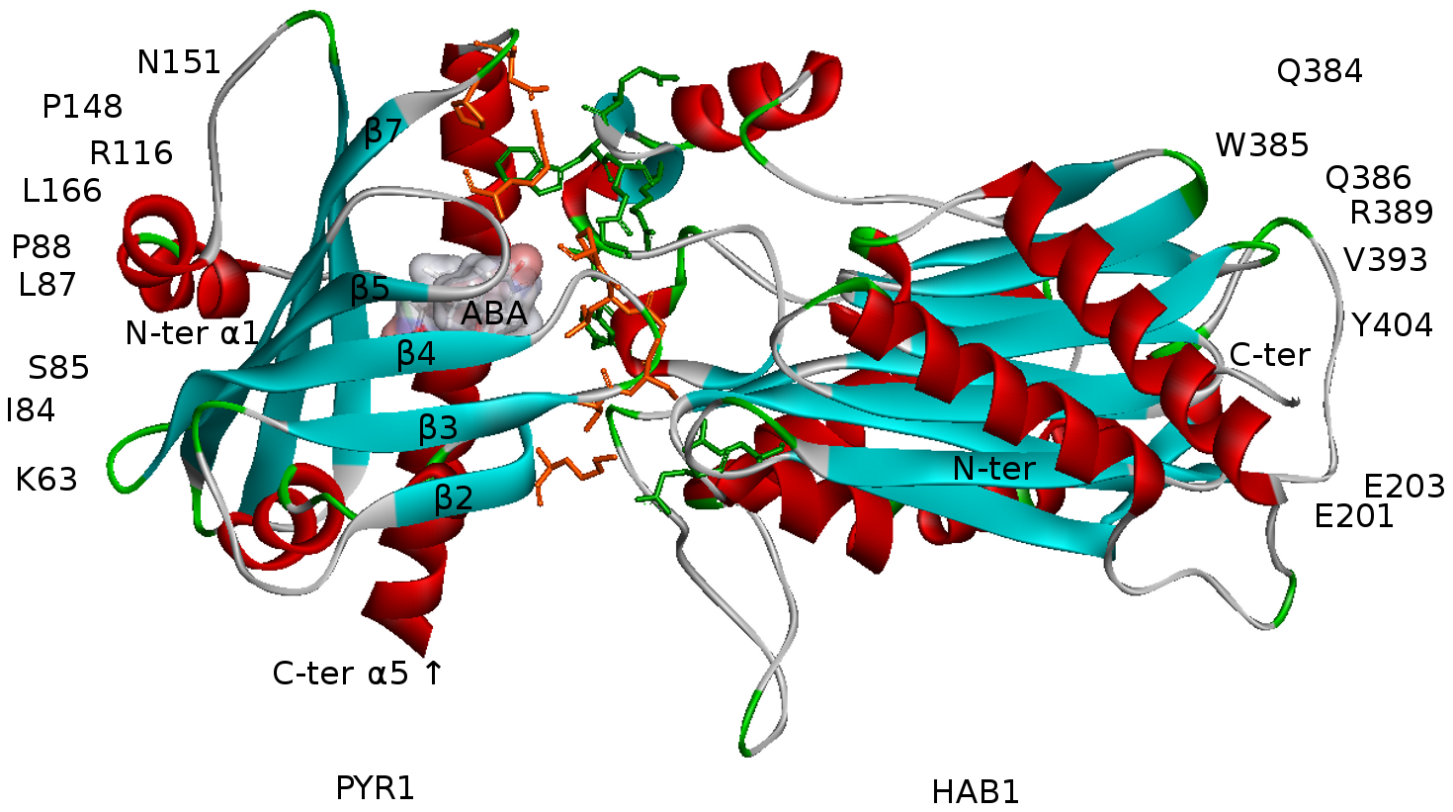
The PYR1-ABA-HAB1 complex (PDB ID 3QN1) with residues on the binding surface shown by orange (PYR1) and green (HAB1) sticks. Polar interactions comprise residues H60–E323, K63–S200, K63–E201, I84–G246, S85–G246, G86–R389, S85–E203, P88–Q386, P88–R389, R116–W385, N151–Q384, and L166–E323. Non-polar interactions include residues F61–Y404, I84–G246, R116–Q386, L87–V393, L117–W385, P148–W385, D155–I383, M158–I383, M158–F391, F159–V393, F159–W385, F159–G392, T142–F391, and L166–Y404 [17]. These include both direct and water-mediated interactions. ABA is represented by a translucent surface, which is colored according to the charge distribution: red for positively, blue for negatively, and white for neutrally charged ABA atoms, respectively.

The structure of ABA-bound PYR1 complexed with homology to ABA insensitive 1 (HAB1) phosphatase (PDB ID 3QN1) is presented in Figure 6. The polar and non-polar interactions on the binding surface indicated in the figure were identified using Accelrys VS, and are consistent with the original structure report [17]. Polar interactions which involve hydrogen bonds correspond to a 4 Å cutoff, and other polar interactions correspond to a 3.5 Å cutoff. The non-polar interactions comprising van der Waals and hydrophobic interactions correspond to a 4.5 Å cutoff.

**Figure 7:**
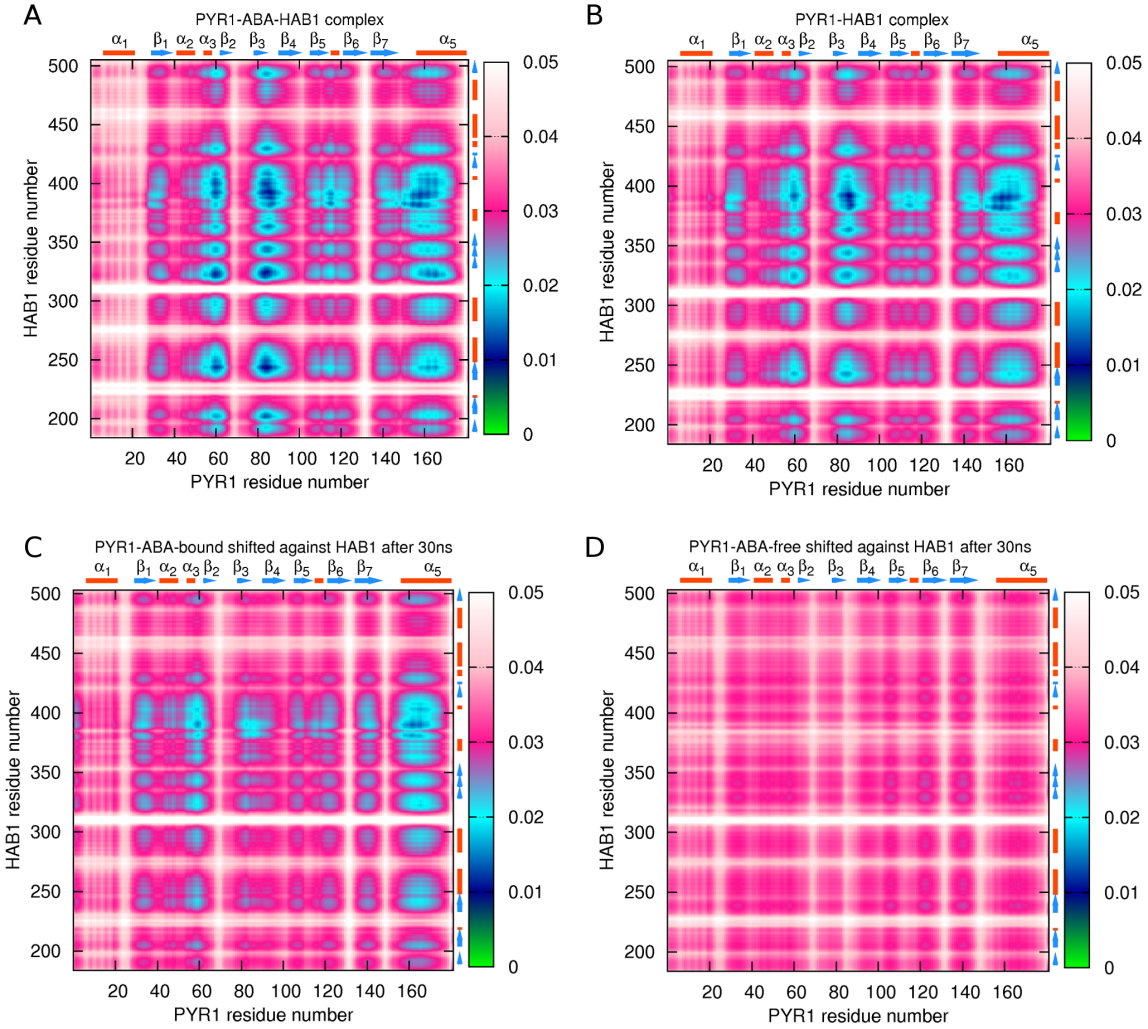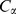 atoms correlation maps for PYR1-HAB1 binding areas: (A) – PYR1-ABA-HAB1 complex; (B) – PYR1-HAB1 complex, ABA extracted; (C) – recovered PYR1-ABA-HAB1 complex in which PYR1 was initially shifted against HAB1; (D) – recovered ABA-extracted PYR1-HAB1 complex in which PYR1 was initially shifted against HAB1. In the maps, lower levels of the correlation descriptor represent strong correlations (green and blue regions), and higher levels correspond a relatively uncorrelated motion (white and magenta regions).

MD simulations conducted for the PYR1-ABA-HAB1 complex over the course of 30 ns simulations showed considerable inter-molecular correlations in the PYR1 and HAB1 binding areas as one would expect (Figure 7 (A)). Simulations for ABA extracted PYR1-HAB1 complex (Figure 7 (B)) demonstrated inter-molecular correlations in similar locations, but with some variability in relative extent. In the ABA extracted complex, N-terminal regions helix α1 and loop Lα1β1 of PYR1 showed a slightly stronger correlation with HAB1 in comparison to the ABA-bound complex, although the level of correlation is slightly lower for the rest of the receptor. Other, minor differences are observed in the areas of loop Lα3β2, the gate, latch, and loop Lβ7α5. In the PYR1-ABA-HAB1 construct these areas show a more pronounced correlation with HAB1, whereas in the case of PYR1-HAB1 complex the loop Lα3β2 has a weaker correlation around residue K63, the gate has reduced correlation around residue G86, correlations for the entire latch are decreased, and loop Lβ7α5 loses the correlations around residue P148. Interestingly, the loop Lα1β1 shows stronger correlations in the PYR1-HAB1 complex than in PYR1-ABA-HAB1 complex, which may be related to disulfide bond formation with C30 as previously reported for residues R157 and C30 [9]. As it has been shown elsewhere, HAB1 residue W385 forms a hydrogen bond with a water molecule, which in turn binds to P88 and R116 of PYR1, and with the ketone group of ABA [17]. It is important that for both constructs A and B of Figure 7, correlations between HAB1 residues around W385 with Lα3β2, Lβ7α5 and the gate are very strong, indicating that tryptophan is inserted in the gate-latch gap independently of the presence or absence of ABA. As it can be seen from close up of PYR1-HAB1 binding area presented on , both ABA-bound and ABA-free closed-lid constructs have a water molecule mediating the interactions of W385 with R116. Other water molecules mediate different important interactions inside of the ligand cavity of both constructs, however the absence of ABA makes these interactions weaker.

**Figure 8:**
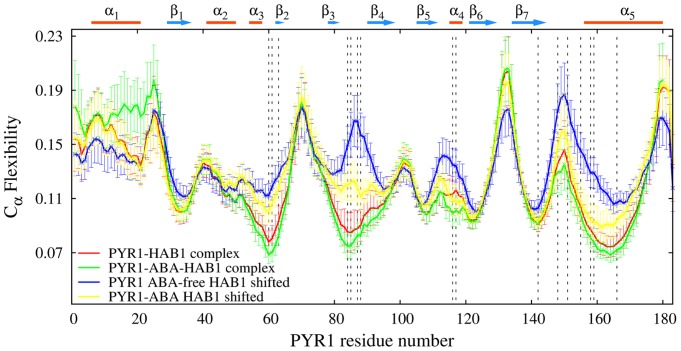
PYR1 main chain flexibility profiles in various complexes with phosphatase: PYR1-ABA-HAB1 complex (green line), ABA-free PYR1-HAB1 complex (red line), partially recovered ABA-bound PYR1-HAB1 complex (yellow line), and partially recovered ABA-free PYR1-HAB1 complex (blue line). The bars indicate the standard deviations. Dashed lines indicate regions of phosphatase binding.

In Figure 8, PYR1 main chain flexibility profiles are presented for both PYR1-ABA-HAB1 and PYR1-HAB1 complexes. Notable is the absence of the flexibility maximum around the gate (which is one of areas of the phosphatase binding) in distinction to the profiles of HAB1-free constructs shown in Figure 3. In agreement with the correlation maps, the flexibility of the latch is somewhat higher in the ABA-free construct than in the ABA-bound construct, indicating than these areas are less constrained in the former case. However, overall the locations of maxima as well as minima of the two flexibility profiles are found at largely similar locations, except for latch residue L117, where a flexibility maxima is observed in the ABA-free construct, but not in the ABA-bound construct. L117 swings outward in the absence of ABA. In conclusion, ABA-bound and ABA-extracted PYR1-HAB1 constructs demonstrate notable similarities in their dynamics, indicating that similar binding mechanisms are likely involved in basal and ligand-induced interactions and suggest that apo-PYR1 should be able to interact with PP2Cs if it is ever free of the homodimer.

**Figure 9:**
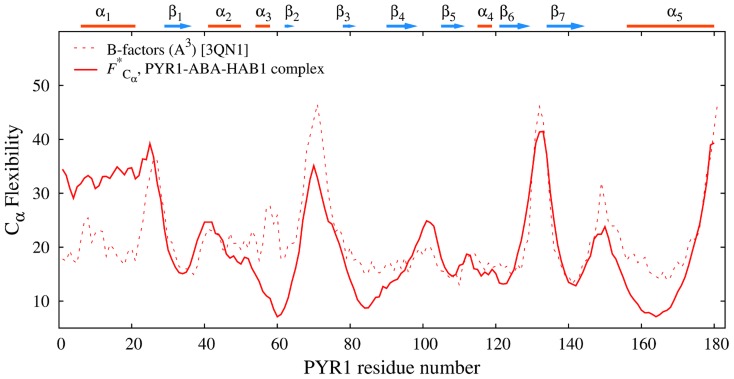
Normalized main-chain flexibility profile 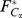 of PYR1 monomer bound to HAB1 (solid lines) over-imposed on B-factors of the starting crystallographic structure 3QN1 (dashed lines).

We also compared the normalized ECD flexibility profiles of HAB1-bound PYR1-ABA construct with the corresponding experimental crystallographic B-factors of the backbone [17] (Figure 9). The two profiles show a reasonable agreement, both exhibiting absence of the flexibility maximum in the area of the gate, as well as a relative decrease of the flexibility in other areas of the phosphatase binding such as the latch and loop Lβ7α5, in comparison to the corresponding profiles of HAB-free monomeric PYR1 in Figure 4. Beyond the region of N-terminus, the most significant difference between the normalized ECD flexibility profile and B-factors is observed in the area of helix α3 and loop Lα3β2. In this area, the values of B-factors show a maximum that is absent in the ECD flexibility, which may be explained by the formation of bonds K59-ABA and H60-S322 observed in the MD simulation.

MD simulations were also performed for both ABA-bound and ABA-free PYR1-HAB1 complexes, in which dissociated closed-lid PYR1 was shifted back against HAB1 for 15 Å, after which 40 ns MD simulations were performed as described in Methods. Snapshots from two independent MD simulations of ABA-bound PYR1 and HAB1 systems are shown respectively in Figures S4 and S5. In one of the MD trajectories (Figure S4), ABA-bound PYR1 has shifted toward the phosphatase during equilibration; the first bond was formed between the gate and residue W385 of HAB1 at 4 ns, and subsequently other connections developed. After approximately 20 ns the entire bond network was recovered. Figure 7 (C) demonstrates the correlations at the binding area after docking has occurred in the first simulation, which bears a significant resemblance with the correlation map for unperturbed PYR1-ABA-HAB1 complex (Figure 7 (A)). In another trajectory (Figure S5) the shift of ABA-bound PYR1 toward HAB1 occurred over the first 8 ns after equilibration, a bond was formed between the gate and W385 followed by development of other connections which also stabilized the folds of HAB1. After approximately 30 ns, PYR1 and HAB1 adopted a similar docking pose as in the initial crystallographic model (PDB ID 3QN1 [17]). With this control experiment in hand, next, we performed similar MD simulations with ABA-extracted (closed-lid) dissociated PYR1 shifted against HAB1. Interestingly, two such simulations again demonstrated binding of apo-PYR1 to HAB1. In one of the simulations, the initially distanced ABA-free PYR1 and HAB1 developed some binding after only 1 ns, however the PYR1-HAB1 interaction remained flexible allowing for a slight rotation and formation of stable bonds after 28 ns. Subsequently, the recovered complex construct remained stable (Figure S6), although the inter-molecular correlations were weaker than in the crystallographic model (Figure 7 (D)). In another simulation, after approximately 20 ns PYR1 and HAB1 have slightly rotated against each other and formed some contacts (Figure S7). In both simulations, the binding surface was somewhat different from that of the complex PYR1-ABA-HAB1.

The PYR1 main chain flexibility profiles for the partially recovered ABA bound and ABA-free PYR1-HAB1 complexes after the initial 15 Å shift are shown in Figure 8. Segments for the ECD analysis were taken from the last 20 ns of the production run, when a recovery of bonds between shifted PYR1 and HAB1 was observed (see Figures S4 and S6). The corresponding flexibility profiles represent constructs with significantly decreased PYR1-HAB1 distances compared to the starting point of the run, approximately from 10 Å to 3.3 Å. For this reason, the levels of PYR1 main chain flexibility (especially in loops Lα1β1, Lβ2β3, Lβ3β4, Lβ6β7 and Lβ7α5 on Figure 8) in the recovered and unperturbed PYR1-HAB1 complexes are significantly lower than in PYR1 receptor alone (see Figure 3). Notably, this decrease of loop flexibility is observed in both ABA-bound and ABA-free recovered complexes, further supporting the possibility that an affinity may be possible between closed-loop PYR1 and HAB1 even in the absence of ABA.

If one compares the four flexibility profiles in Figure 8, it is clear that the flexibility is overall lower in both unperturbed (non shifted) simulations, in particular in the areas of the loop Lα3β2, the gate and helix α5. Indeed the regions around the gate, the loop Lβ7α5, the C-terminal helix α5 (residues D146-L171), as well as residues K54-S66 all show a lower flexibility in unperturbed PYR1-ABA-HAB1 complex than in both recovered constructs. This is further emphasized by the higher flexibility in the recovered ABA-free complex, in particular around residues I62, G86 (near the gate), H115, R116 (near the latch), and E149, S152, W156, A160 (in Lβ7α5 and helix α5). Interestingly the unperturbed PYR1-ABA-HAB1 complex does have higher flexibility around residues F20 (loop Lα1β1) and E132 (loop Lβ6β7), but these are both relatively distant from the binding area. The flexibility of recovered PYR1-ABA-HAB1 complex is lower than that of recovered ABA-free PYR1-HAB1 complex by 35% around residue M158 in loop Lβ7α5 and by 42% around G86 in the gate. This indicates that a significant constraining of the loop Lβ7α5 as well as the gate occurs in the presence of ABA in the recovered system.

Complementary to the ECD analysis, nearest inter-molecular neighbors identified by Accelrys VS with a 5 Å cutoff have also been compared for the initial PYR1-ABA-HAB1 complex and the recovered PYR1-ABA-HAB and PYR1-HAB complexes (see Table S1, blocks I, II, and III, respectively). Nearest inter-molecular neighbors for the PYR1-ABA-HAB1 construct with mutation H60P (PDB ID 3ZVU, [16]), PYL2-ABA-HAB1 complex (PDB ID 3KB3 [11]), PYL3-ABA-HAB1 complex (PDB ID 4DS8 [38]), and ABA-free PYL10-HAB1 complex (PDB ID 3RT0, [15]) are also listed in Table S1 (blocks IV-VII, respectively). It can be seen that the binding interfaces of receptors in constructs 3QN1, 3ZVU, 3KBS, 4D58, and 3RTO are highly conserved and include residues of Lα3β2, the gate, the latch, Lβ7α5 and helix α5 (F61, S85-P88, R116, N151, D155, F159, T162, L166). The recovered PYR1-ABA-HAB1 complex exhibits largely similar binding areas, in agreement with the described ECD analysis. In the recovered ABA-free PYR1-HAB1 complex, an extended Lβ3α2 area, some residues of the gate and helix α5 also developed bonds with the phosphatase. Further to this, PYR1 regions around the loop Lβ6β7 (E132-R134), C-terminal region of α5 (R180), and the N-terminal region of Lβ3α2 (P55-K59, not shown in Table S1) developed bonds with HAB1 only in the recovered ABA-free PYR1-HAB1 complex and not in the other constructs. It can also be seen that the regions of the phosphatase involved in the binding of the recovered ABA-free PYR1-HAB1 complex are somewhat different from those in the initial PYR1-ABA-HAB1 construct (3QN1) as well as in the other PYR and PYL constructs listed in Table S1.

Overall these results highlight some of the dynamical aspects of the PYR1 structure that are important in mediating PYR1-HAB1 complex formation; highlighting some ABA-dependent differences in the roles of loop Lα3β2, the gate, the latch, loop Lβ7α5, and helix α5. These findings are consistent with previous crystallographic and mutagenic reports, and also support the possibility of the formation of functionally relevant ABA-free PYR1-PP2C interactions. On the other hand, MD simulations demonstrate that the absence of ABA decreases the aptitude of PYR1 to quickly develop proper contacts with the phosphatase. This supports the possibility of some low level basal PYR1 activity in association with these interaction kinetics.

This computationally predicted dynamical data begs the question of whether binding of PYR1 to PP2Cs occurs only in the presence of ABA or whether select concentrations of receptors and phosphatases might enable an ABA-free PYR1-HAB1 interaction. While there is no substantial evidence for such an apo-PYR1-PP2C interaction in the literature to date, one fairly systematic analysis of the basal activity of the ABA receptors against four different PP2Cs did reveal up to ∼45% inhibition of HAB1 activity by apo-PYR1 at an RCAR∶PP2C ratio of 100∶1 at 0.27 µM HAB1 [15]. However a different study [39] working with a 4∶1 ratio was unable to detect any inhibition of PP2CA by PYR1 and only a weak inhibition by PYL8 was observed. Further detailed evaluation by *in vitro* experiments in our own lab, in which freshly purified recombinant PYR1 was titrated into the phosphatase ABI2 across a range of protein ratios showed a maximal basal effect at a 4∶1 receptor∶phosphatase ratio (up to 80% inhibition of ABI2 activity) at a constant concentration of 0.5 µM ABI2 (Figure S8). It is interesting that this interaction seems to be conditional, occurring over a relatively narrow range of RCAR∶PP2C ratios starting at around equimolar and peaking at 4∶1 and then decreasing at higher ratios. The decrease at higher ratios would be consistent with increased homo-dimerization of PYR1, sequestering it away from the PP2C. The potency of the apo-PYR1-ABI2 interaction reported here, while at odds with those published previously [15], [16], may relate to subtle differences in the actual protein concentrations tested and the identity of the PP2C. Hao et al., [15] only see potency of apo-PYR1 against HAB1, and not ABI1, HAB2 or PP2CA (they do not provide data for ABI2 at all). As well the freshness of the protein preparation may have an impact, as freeze storage of at least one PYL has been shown to selectively abolish basal signaling functionality of the receptor, without affecting its ABA-induced activity (Figure S9). Overall, these relative activities for the basal PYR1-ABI2 versus PYR1-ABA-ABI2 interactions (compared directly at a 1∶1 RCAR∶PP2C ratio (Figure S8) as well as the apo-PYR1-HAB1 interaction described previously [15], are consistent with the dynamic variations described above with increased correlations and more stable profiles detected for the PYR1-ABA-HAB1 complex in the areas of loop Lα3β2, the gate, latch, and loop Lβ7α5.

### Comparative ECD Analyses of PYR1-PYR1 and PYR1-HAB1 Systems

That the low or complete lack of basal activity reported for dimer-forming receptors including PYR1/PYLs 1–4, may result from a ‘competitive interaction’ process between homo-dimer complexes and receptor/phosphatase complexes has been put forward in several publications [7], [15], [16]. The results reported in the previous section support the possibility that PYR1-HAB1 binding is possible in the absence of ABA, lending further support to the ‘competitive interaction’ mechanism for PYR1. Vice versa, our observation that repeated freezing abolishes the basal activity of a ‘monomeric’ receptor (PYL5; see Figure S9), but not its ABA-inducible activity, suggests that under certain conditions, it may even be possible for these monomeric receptors to form homo-dimers (oligomers).

To date, published experimental studies have shown that loops Lα3β2, as well as the gate, the latch and the loop Lβ7α5 are involved in determining the outcome of the competition [9], [16], [17]. In particular the importance of loop Lα3β2 was demonstrated in experiments by Dupeux et al [16], which revealed that residue H60 may determine the oligomeric state of PYR/PYL family members. In turn, conformations of the gate, the latch and the loop Lβ7α5 (often denoted as Pro cap, Leu lock and partially “Recoil motif” subunits) have been shown to be somewhat different for ABA-bound and ABA-free PYR1 asymmetric dimers [9]. A subtle difference of conformations around residue S85 and Lβ7α5 loop between ABA-bound dimer and PYR1-ABA-HAB1 also indicate that these regions indeed play a role in the binding of PYR1 and HAB1 [17]. However, the dynamical contributions of these regions to determining the selectivity of interactions remain to be explored by molecular simulations. Thus we extended our study to address PYR1 dimers in comparison with the PYR1-HAB1 complexes in the presence and absence of ABA.

**Figure 10:**
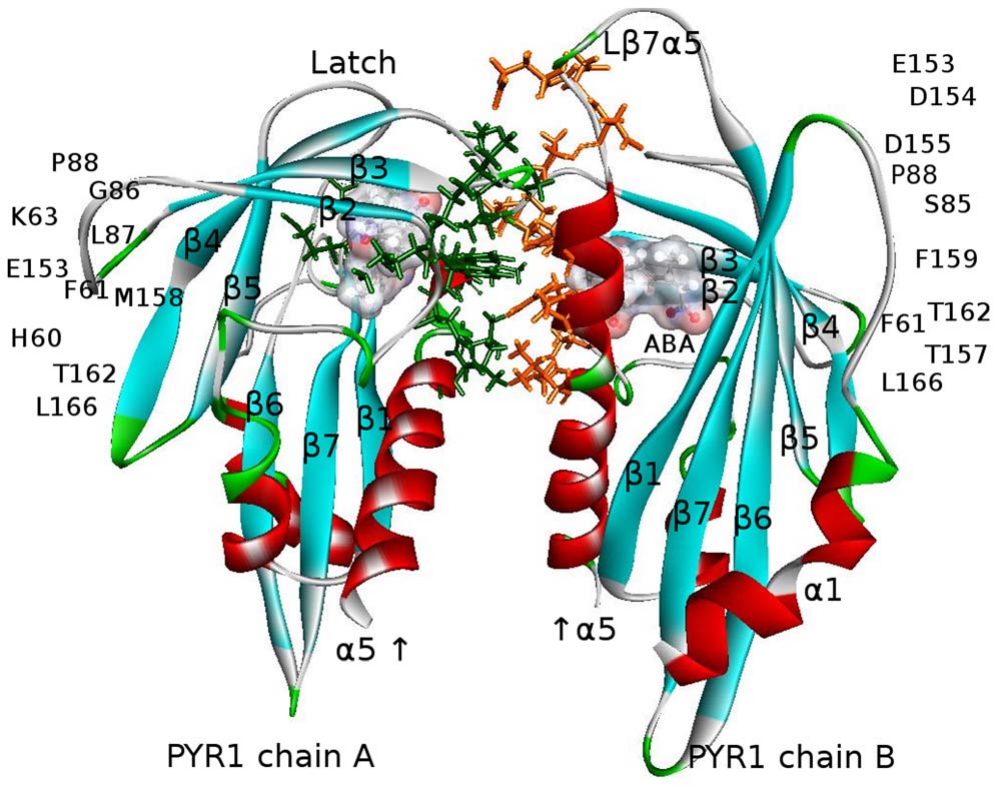
PYR1-dimer, 2ABA -bound (modified PDB ID 3NJO after ligand replacement, mutation S88P, minimizations and equilibrations in water) with residues on the binding surface indicated by orange sticks for chain A and green sticks for chain B. The direct and water-mediated interactions, detected by AccelrysVS employing the same criteria as for the PYR1-ABA-HAB1 complex in Figure 6, comprise H60–L166, H60–T162, F61–F159, F61–L166, F61–F61, F61–T162, I62–M158, K63–D155, K63–E153, I84–F159, S85–D155, S85–D154, S85–F159, S85–T156, S85–E153, G86–P88, G86–L87, G86–F159, L87–P88, L87–L87, L87–F159, P88–P88, L166–L166 and all reciprocal [10]. Ligand molecules in the binding pockets are depicted by surfaces colored according to the charge distribution as in Figure 6.

Studies have shown that PYR1 forms homodimers in the absence of ABA [15], [16], or possibly with ABA occupying one binding site between the two dimer partners [9]. On the assumption that the presence of two ABA molecules in a dimer leads to monomerization, simulations on a PYR1 dimer construct containing two ABA ligands, which is denoted as the 2ABA-bound dimer, were initiated. This construct has been prepared from PDB structure 3NJO, where the pyrabactin (PYV) and P2M ligands were replaced with ABA molecules. The structures of symmetric ligand-free dimers, and occasionally, the asymmetric 1ABA-bound dimer have also been used for comparison (see Table 1 and Methods). Because the available PDB crystallographic structure of PYR1 dimer contains PYV/P2M ligands, we also investigated the binding of these ligands along with ABA. The structure of a 2ABA-bound dimer after equilibrations in water is shown in Figure 10. The identified interactions are in agreement with published structural data [9], [10]. As previously reported, the regions of PYR1 involved in binding to HAB1 (Figure 6) and in the dimer (Figure 10) overlap significantly [17].

**Figure 11:**
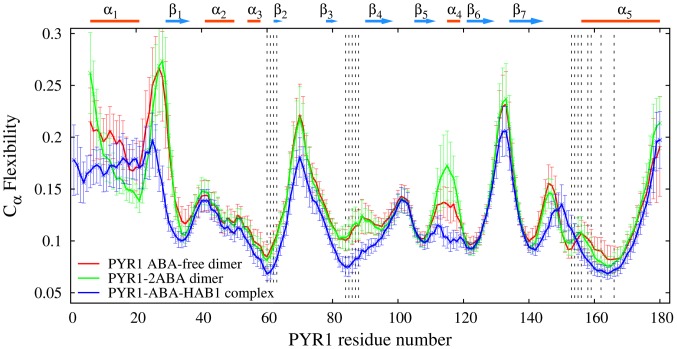
PYR1 main chain flexibility profiles in ABA-free PYR1 dimer (red line), 2ABA-bound PYR1 dimer (green line), and ABA-bound PYR1 in complex with HAB1 (blue line). Overall, the main chain of PYR1 is more flexible in the dimers than in the PYR1-HAB1 complex. Dashed lines indicate the regions of dimer binding. The level of PYR1 flexibility in the dimer/HAB1 complex is essentially reduced comparing to that of PYR1 monomer (Figure 3).

The MD simulations for the dimers have been carried out for 30 ns after a 10 ns NPT equilibration. Figure S10 shows the main chain flexibility profiles for the PYV/P2M- and 2ABA- bound PYR1 dimers, as well as for ABA-free apo-dimer, whereas Figure 11 compares the flexibilities of PYR1 from ligand-free and 2ABA-bound PYR1 dimers with that from the complex PYR1-ABA-HAB1. These comparisons demonstrate that in most regions, flexibility and its standard deviation are higher in ABA-bound PYR1 dimer (both 2 ABA and 1 ABA forms) than in any other complex. In particular, the flexibility in the latch region (H115) of 2ABA-bound and ABA-free dimers is higher than in PYR1-ABA-HAB1 complex by 67% and 27%, respectively. We attribute this difference to the effect of bonds L117-W385 and R116-Q386 in the PYR1-ABA-HAB1 construct. In ABA-free dimer, residues H115 in both chains are asymmetrically involved in intra-receptor binding with D154 and A89 each, whereas in ABA-bound dimers residue P88 interacts only indirectly with ABA, making the gate and latch areas more flexible and also destabilizing helix α4. Interestingly, the increase of flexibility around residue H115 is more pronounced in the 1ABA-bound-dimer than in either the 2ABA-bound dimer or the pyrabactin-bound dimer (Figure S10). In the PYV-bound dimer, H115 can interact with A89 because of the smaller size of PYV size and residue 88 tends to interact with the latch rather than with the ligand, stabilizing helix α4. As it also can be seen from Figure S10, binding of PYV/P2M or two ABA ligands decreases the flexibility of helix α5 in comparison with ligand free dimers. Another notable feature is observed in the loop Lβ7α5 and around residue A89. In the first region, dimerized PYR1 develops bonds at residue E153, whereas in the PYR1-HAB1 complex, the binding occurs at residues N151-Q384 and P148-W385 which causes a slight shift in the flexibility maximum in the region of this loop. These differences between dimerized and phosphatase-bound PYR1 constructs agree with the recent analysis of corresponding crystallographic data [17]. Finally, for all dimer models considered, a relatively high flexibility for loops Lα1β1 and Lβ2β3 is observed since these loops are dynamically uncoupled from the rest of the construct.

**Figure 12:**
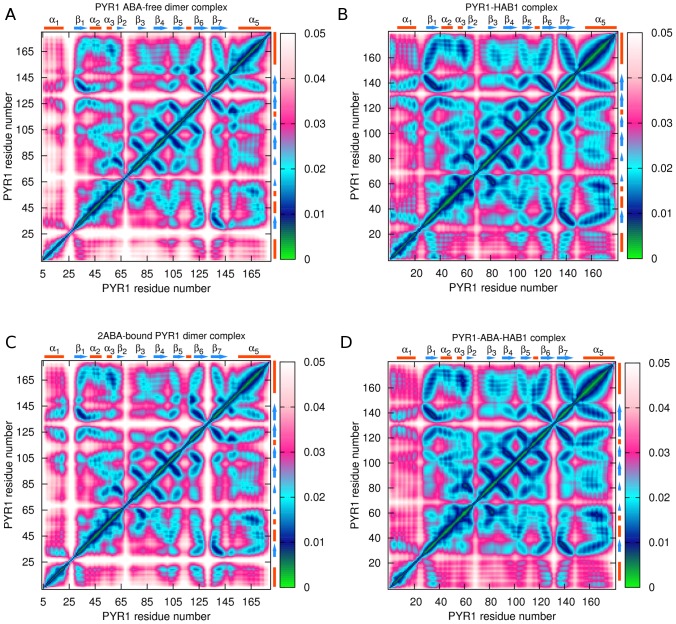
Intra-receptor 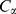 atoms correlation maps: (A) – in ligand-free PYR1 dimer; (B) – in PYR1-HAB1 complex; (C) – in 2ABA-bound PYR1 dimer; (D) – in PYR1-ABA-HAB1 complex. Strong correlations are represented by low values of the descriptor (green and blue colors), whereas high values indicate a more independent motion (magenta and white colors).

**Figure 13:**
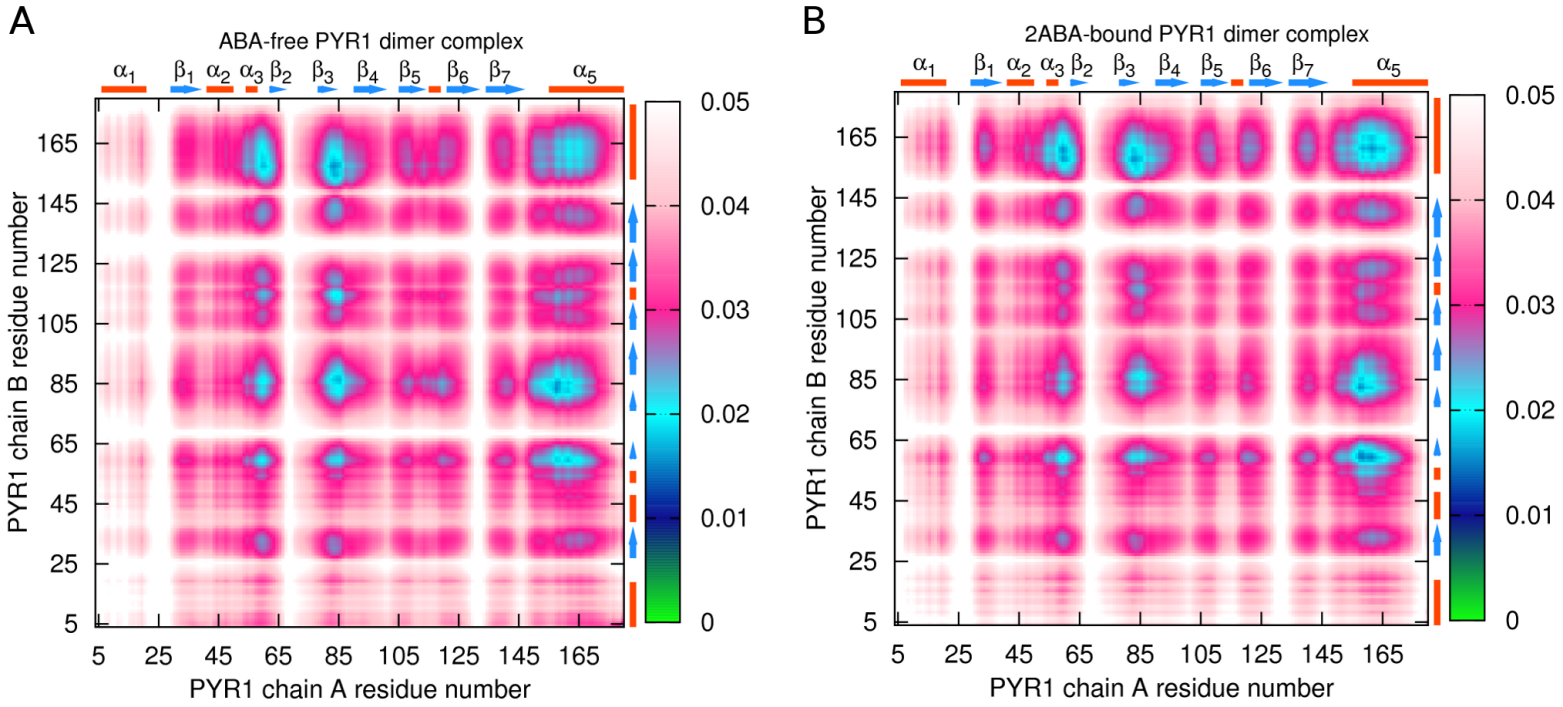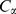 atoms correlation maps for binding area between A and B PYR1 chains in ligand-free dimer (A) and 2ABA-bound dimer (B).

Main chain correlation maps for the dimer constructs were subsequently analyzed. Intra-receptor correlations for 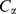 atoms in ABA-free dimer and PYR1-HAB1 as well as 2ABA-bound dimer and PYR1-ABA-HAB1 complex are shown in Figure 12. It is clear from comparison of Figure 12 panels (A) and (B) that intra-receptor correlations are overall weaker in the ABA-free PYR1 dimer compared to the ABA-free PYR1-HAB1 complex. However, in 2ABA-bound dimer intra-receptor correlations for the latch region and loop Lβ4β5 are even weaker still, supporting a model in which 2ABA-binding destabilizes apo-homodimer complexes. In contrast, the presence of ABA leads to stronger correlations in the ABA-bound PYR1-HAB1 (Figure 12 D) complex than in either dimer or the apo-PYR1-HAB1 complex. Especially evident is the relative lack of intra-molecular correlations in the area of the C-terminal helix α5 in both apo- and holo- dimers (Figure 12 A and D). As well, helix α5 coupling to residues 30–62, gate, latch and β7 is approximately three times weaker in the dimers than in the PYR1-ABA-HAB1 complex. Inter-molecular correlation maps of the binding areas of ligand-free and 2ABA-bound dimers shows a similar inter-correlation network in the two constructs (Figure 13). One difference is that the residues from the latch region in chain A of the 2ABA-bound dimer show less correlation with chain B generally. Also, the coupling of gate residues is slightly weaker with ABA in the dimer. Overall, the inter-molecular correlations are slightly less symmetrical for the 2ABA-bound dimer, arising from the observation that correlation of the chain A gate with the chain B helix α4 is not mirrored by a similar correlation between chain B gate and chain A helix α4 in agreement with published X-ray scattering experiments of assymetric dimeric units [9].

Comparison of Figures 13 and 7(A) confirms that similar regions of PYR1 are involved in HAB1 and dimer interfaces. In particular, residues K54-S66, R79-W93, and D146-L171 are involved in both dimer association and HAB1 binding. However, in most of these areas PYR1 residues are less constrained in the dimer than in the PYR1-HAB1 complex. Thus, a relatively weak coupling of chains A and B of the dimer is observed in the region of residue H60, the gate, N-terminal of helix α5, and to a lesser extent in the latch and C30 region. These differences originate from the interactions of the latch region and T142-N151 region residues. While the latch region is strongly correlated with several large parts of the phosphatase, pronouncedly weaker correlations are observed in the dimer, particularly around residue 111. A similar trend of decoupling is observed at residue 145 of the dimer.

### Conclusions

In an effort to elucidate details of the molecular mechanisms mediating PP2C inhibition by ABA receptors, we report extensive molecular dynamics simulations of apo and holo pyrabactin receptor PYR1 in complex with HAB1 as well as in dimeric form. We also report our comparative analysis of the dynamical stability of these complexes by novel ECD method, which we have validated against crystallographic B-factors.

In agreement with recent experimental findings [8]–[16], our MD simulations and the ECD analysis indicate that ABA-bound PYR1 should efficiently bind to HAB1. In particular, the loop Lα3β2, the gate, latch, loop Lβ7α5, and helix α5 have been found to develop stronger dynamic correlations with HAB1 in presence of ABA in comparison to ABA-free constructs. However, ABA-bound and ABA-extracted PYR1-HAB1 constructs demonstrated notable similarities in their dynamics, suggesting that apo-PYR1 should be able to make a substantial interaction with PP2Cs. This possibility was validated by *in vitro* data that demonstrate a conditional functional interaction between apo-PYR1 and ABI2 in our hands. In the context of competing interactions, our dynamical analysis indicates that although similar regions of PYR1 are involved in dimer association and HAB1 binding both ABA-bound and ABA-free PYR1 in complex with HAB1 exhibit a lower flexibility, higher intra-molecular structural stability, and stronger inter-molecular dynamic correlation, in comparison with either holo- or apo- PYR1 in dimeric form. This may be interpreted as dimeric PYR1 being under less steric constraint in comparison with PYR1-HAB1 complex. Furthermore, comparison of 2ABA-bound and ABA-free dimers reveals a loss of intra-receptor correlations, in particular in the areas of the latch and loop Lβ4β5, upon ABA binding. Inter-chain correlations in the area of the latch and the gate are also somewhat weakened in the presence of ABA.

Together these results are consistent with ABA having an opposite effect on PYR1-HAB1 and PYR1-PYR1 complexes, constraining the former and destabilizing the latter, as expected. They also suggest that ABA-free PYR1 can bind to the phosphatase, and that such binding would be in competition with PYR1 dimerization, particularly in the absence of ABA. These findings, validated *in vitro*, suggest that the model of receptor regulation by ‘competing interactions’ may be more complex and at the same time more broadly applicable to all PYR1/PYL type ABA receptors both in the presence and absence of ABA. Finally, these findings raise the question of whether the dynamics of receptor-phosphatase interactions might be preferred over that of homo-dimer interaction regardless of the presence or absence of ABA in the PYR1 binding pocket, and whether the ‘competing interactions’ mechanisms could, to a significant extent, be regulated by kinetic factors such as the interaction reaction pathway or the availability of protein [39].

## Methods

### Molecular Structures

Crystallographic coordinates of the pyrabactin receptor PYR1, as well as the complexes PYR1-ABA-HAB1 and PYR1-pyrabactin-bound dimer (resolution 1.70 Å, 1.80 Å and 2.47 Å respectively) were taken from the Protein Data Bank [36], entries 3K3K, 3QN1, and 3NJO [9], [10], [17], see Table 1. Homology to ABA insensitive 1 (HAB1) phosphatase model was chosen based on the availability of the 3D structure in complex with PYR1. Initially, all water molecules were removed from the crystal structures. All ligands extractions/insertions were created *in silico* with Accelrys Discovery Studio software [40]. In structure 3QN1, 26 missing residues, G222-L231, D271-R282, and P462-E465 and missing atoms in residues 214, 281, 233, 406, 422, 468, and 504 were reconstructed using a replacement structure 3RT0.pdb, chain A of apo-PYL10-HAB1 complex [15]. Missing residue P229 was built with Accelrys DS. For the ABA molecule, Accelrys DS was used to add the hydrogens, and acpype [41] and mktop [42] scripts were employed to evaluate the charge distribution and generate topology files. All constructed regions were optimized.

As a model of monomeric ABA-bound closed lid PYR1 construct, chain B of structure PDB ID 3K3K was used, whereas chain A of 3K3K was used to model open lid ABA-free PYR1 construct. To simulate ABA-free closed lid PYR1 construct, ABA was extracted from chain B of 3K3K (see also Figures S1 and S2). All constructs were minimized *in vacuo*, and solvated in water with counterions afterwards.

The model of ABA-bound PYR1 in complex with HAB1 employed structure PDB ID 3QN1, and the ABA-free PYR1-HAB1 structure was obtained by extraction of ABA from 3QN1 and further minimization and equilibration. The same crystallographic model 3QN1 was used to construct ABA-bound and ABA-extracted PYR1-HAB1 systems, in which PYR1 was initially shifted against HAB1 (see Figures S4, S5, S6, S7). To prepare a separated ABA-bound construct, the centers of mass of PYR1 and HAB1 were oriented along the 0Z axis, and then the coordinates of ABA-bound PYR1 receptor were shifted against the phosphatase for 15 Å along the 0Z axis using Accelrys DS [40]. After *in vacuo* minimization of the ABA-extracted PYR1-HAB1 complex, similar to described above centers of mass alignment has been made, then the receptor was shifted against HAB1 for 15 Å along the 0Z axis.

The PYR1 dimer constructs were built from PDB ID 1NJO structure. In this model, subunits of the P88S mutant dimer contain synthetic ligands C_16_H_13_BrN_2_O_2_S (pyrabactin, PYV) and C_16_H_14_N_2_O_2_S (P2M), and the mutation P88S is introduced to improve binding of these ligands. To construct PYR1 models containing one and two ABA ligands, PYV/P2M ligands were extracted from 1NJO and replaced with one or two ABA molecules respectively, after which reverse mutation S88P has been made using Accelrys DS, and minimization was performed. In the paper, dimers containing one and two ABA molecules are denoted as 1ABA-bound and 2ABA-bound dimers, respectively. In the model of ligand-free dimer, PYV/P2M molecules were extracted from structure 3NJO, residue S88 replaced with P88, and minimization performed. For simulations involving PYV-bound PYR1 monomers and PYV/P2M bound dimers, residue S88 was retained, structure minimized and solvated.

After *in-vacuo* minimization, each system was solvated in a triclinic box with walls located at distances ≥15 Å from the protein. Simple Point Charge (SPC) water molecules were employed, and Na^+^ or Cl^−^ counterions were added to make system net charge equal to zero.

### Molecular Dynamics Simulations

Minimizations, equilibrations and production MD simulations were carried out using Gromacs v4.0.7 [43] and AMBER v11 packages [44] with OPLS and AMBER03 force fields, respectively. The trajectories generated by Gromacs were used to analyze structural changes as well as for ECD analysis.

*In vacuo* minimization of starting models described in the previous section comprised 10000 steps of steepest descent minimization. After the systems were solvated, solvent minimization was made using 500 steps of a steepest descent algorithm with strong positional restraints on all heavy protein atoms to prevent distortion of protein structure by non-equilibrated solvent. Next, seven steps of short steepest descent minimization were performed on each solvated system with decreasing position restraints on non-hydrogen protein atoms (K_posre_ = 1×10^5^, 1×10^4^, 1000, 100, 10 and 0 kJ mol^−1^ nm^−2^) followed by system heating. The temperature of proteins and solvent was maintained at desired level (310 K in most cases) by coupling the systems with Berendsen thermostats [45]. Seven NVT-like MD equilibration steps with decreasing non-hydrogen protein position restraints (K_posre_ = 1×10^5^, 1×10^4^, 1000, 100, 10 and 0 kJ mol^−1^ nm^−2^) were then made, the last one with no restraints and stronger bath coupling. The last equilibration step and production simulations were conducted at the desired temperature of 310 K (unless indicated otherwise) and pressure at 1 atm with isotropic pressure coupling (NPT ensemble), bond length restrained with the LINCS algorithm with a fourth order of expansion. The short-range electrostatic and van der Waals interactions cutoff radii were equal to 14 Å each. Long-range electrostatic interactions were treated with particle-mesh Ewald (PME) summation with grid spacing 0.135 nm for the fast Fourier transform and cubic interpolation.

In order to validate our model building protocol, we have computed the root-mean-square deviations (RMSD) between main-chain atoms of asymmetrical one-ABA-bound dimer construct (PDB ID 3K3K [9]) and a similar model structure prepared from modified 3NJO [10] construct where S88P mutations were introduced in both chains, pyrabactin and P2M extracted, and one ABA ligand inserted. After minimization of the modified structure, 6 heating steps of 50 ps each were performed followed by a 450 ps NVT equilibration, and subsequently by a 200 ps NPT equilibration. The coordinates of the modified 3NJO construct were subsequently aligned with the reference 3K3K construct, and the corresponding RMSDs were computed using VMD software [46]. For the modified 3NJO structure, core domains (33–37, 53–57, 59–65, 80–84, 90–94, 105–108, 110–112, 121–127, 134–141, 158–162, 166–168, 170–172) were employed to obtain the backbone (C, Ca, N) RMS deviations. Both the initial crystallographic 3K3K structure and the solvated and equilibrated 3K3K construct were employed as references. When using the original crystallographic structure 3K3K as a reference, the RMSD after NVT equilibration were between 0.32 and 1.32 Å. Figure S11 illustrates the evolution of the RMSD during last stages of NVT equilibration and also during NPT equilibration. Over the first 200 ps long process of NVT equilibration seen in the figure (400 ps–600 ps), the constraints applied kept the RMSD stable around 1.34 Å, whereas on the interval from 600 ps to 750 ps the constraints were released and backbone atoms have readjusted resulting in RMSD levels fluctuating between approximately 1.05 Å and 1.45 Å. Over the following 200 ps NPT equilibration was performed, during which RMSD first increased from approximately 1 Å to an average of about 1.25 Å and then stabilized. Figure S12 shows the secondary structure alignment of the modified 3NJO construct and the crystallographic 3K3K model after completion of the NPT equilibration right before the production run.When a solvated and equilibrated 3K3K model was used as a reference, the corresponding RMSD were between 0.26 and 1.23 Å.

The production MD simulation runs were performed from 20 ns to 50 ns depending on the system with 1 fs time steps, and snapshots saved every 20 fs in order to analyze the essential collective dynamics. The ABA-bound PYR1 closed lid system was simulated for 40 ns at 300 K and for 30 ns at 325K, the ABA-free PYR1 closed lid systems were simulated for 20 ns at 281 K, for 50 ns at 300 K, for 20 ns at 310 K, and for 30 ns at 325K; and ABA-free open lid PYR1 construct was simulated for 30 ns at 281K and for 30 ns at 300 K, using the Gromacs MD simulation package. The ABA-bound and ABA-free PYR1-HAB1 complexes, and PYR1 dimer complexes were simulated for 30 ns at temperature of 310 K. For ABA-bound and ABA-free complexes with PYR1 shifted against HAH1, two independent molecular dynamics (MD) simulations of 40 ns each have been carried out.

### Essential Collective Dynamics of Proteins

The ECD method relies upon a recently developed statistical-mechanical framework [31]–[34], according to which a macromolecule can be described by a set of generalized Langevin equations (GLE) with essential collective coordinates, which can be deduced by applying PCA on MD trajectories. The latter procedure provides a set of principal eigenvectors of the covariance matrix 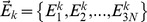, 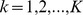. Here 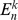 represent the direction cosines of the eigenvectors, where 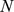 is the number of atoms in the system, and 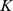 is the number of eigenvectors which sample a sufficient percentage of the total displacement, which are often referred to as essential collective coordinates. Usually, 10–30 essential coordinates are sufficient to sample approximately 90% of the displacement for a typical MD trajectory of a protein. In the ECD method, an all-atom projected image of the protein is constructed in the 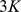 dimensional space of essential collective coordinates such that the position of each atom is characterized by 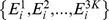, where 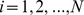. The theory shows that such an image represents the degree of dynamic correlation (coupling) between the protein's atoms: points (images of atoms) that are located close to each other correspond to atoms whose motions are strongly correlated regardless of their proximity in secondary or tertiary structure of the protein, and more distant points correspond to a relatively independent motion [31], [33]. A suite of simple structural descriptors, such as the main chain flexibility and domains of correlated motion, have been derived within the ECD framework and successfully employed to analyze dynamics of proteins [32], [35], [47], [34]. It has been both proven theoretically [33] and confirmed by comparing numerical predictions with NMR experiments representing microsecond time regimes [31], [34].

In this work, the dynamics of PYR1 constructs were characterized primarily employing ECD derived correlation maps and flexibility profiles. The ECD correlation maps are distances between images of atoms in the 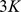 dimensional space of essential collective coordinates. These distances are dimensionless quantities represented by [34]

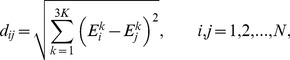
(1)with lower values of 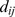 representing stronger correlations. In this work, Equation 1 has been employed to visualize correlations between 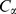 atoms both within PYR1 molecules and across molecules in PYR1 dimers as well as complexes with HAB1 atoms in order to examine the corresponding intermolecular and intramolecular dynamics.

While ECD derived flexibility profiles allows characterizing the flexibility with atomic-level precision, a per-residue flexibility assessment is sufficient in many cases [32], [34]. Here, the flexibility for 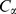 atoms in the main chain of the various PYR1 constructs was analyzed. The ECD flexibility descriptor 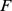 for a 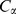 atom in residue 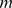,
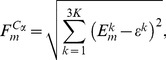
(2)defined by the distance in the 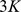 dimensional space of essential collective coordinates between the image point representing the 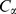 atom and the centroid calculated over the images of all 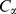 atoms,
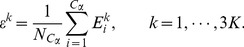
(3)In Equation 3, 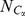 is the total number of 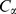 atoms in the molecule. By definition, the ECD flexibility descriptor 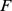 represents the level of dynamic coupling of the motion of individual 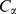 atoms with the entire molecule, which in turn is represented by the centroid of all 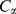 atoms in the space of essential collective coordinates.

For each construct considered, we employed the ECD analysis with 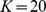, on 100 segments, each of 20 fs, from the last 20 ns of MD trajectories.

Finally, the ECD framework also allows identifying dynamic domains of correlated motion, which represent relatively rigid parts of the protein composed of atoms moving coherently. Such domains can be identified through a simple nearest-neighbor clustering of the protein's image in the space of essential collective coordinates, as described in detail elsewhere [31], [32], [35], [34]. When employing the nearest-neighbor clustering technique to identify the dynamic domains, an interdomain distance 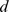 must be selected which represents the minimum degree of correlation for two atoms to belong to the same domain. In this paper 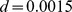, has been adopted as an optimum value, which maximizes both the domains number and the difference of total number of atoms in all domains and number of atoms in the largest domain, as well as includes reasonable (more than 10%) average number of atoms in the largest domain. A further discussion of the choice of the interdomain distance can be found elsewhere [31], [32], [34]. An extensive series of test MD simulations for PYR1, both closed lid and open lid constructs, as well as ABA and pyrabactin bound and ABA-free, at various temperatures for 20 to 50 ns was carried out.

## Supporting Information

Figure S1 Dynamical domains of correlated motion for the pyrabactin receptor (PYR1), closed lid and ABA-extracted. Simulations were performed at (A) 281 K, (B) 300 K, (C) 310 K, (D) 325 K. Interdomain distance d = 0.0015 has been adopted. The color scheme is as in Figure 4. The largest domain, coloured blue, indicates the most extensive dynamical correlations.
(TIFF)

Figure S2 PYR1 closed lid, ABA extracted construct after 20 ns of simulations at 300 K (A), 310 K (B), and 325 K (C). In (A) structure retains the closed lid conformation, in (B) the gate and latch have decoupled, however the contact of L87 with M158 is observed in approximately 30% of simulation snapshots; in (C) the gate detached from the latch and helix α5.
(TIFF)

Figure S3 Close up of the binding area for ABA-bound (A) and ABA-free (B) PYR1-HAB1 complexes obtained from MD simulations using Accelrys VS. ABA and the residues W385, R116 and P88 are shown in orange, green, light grey and dark grey sticks, respectively. The water molecules are shown as red-and-white sticks.
(TIFF)

Figure S4 Snapshots from MD simulation of closed lid, ABA-bound PYR1 and HAB1, initially shifted away from each other by 15 Å, at 310°K (different trajectory than in Figure S2): Immediately after minimizations and equilibrations (A), and after production run of 4 ns (B), 13 ns (C), 18 ns (D), and 20 ns. In (A), the distance from PYR1 to HAB1 is decreased to 5 Å; in (B), binding of HAB1 to helix α5 of PYR1 (M158-F391) occurred; (C) captures HAB1 slowly approaching the rest of binding surface; in (D), more bonds are formed (G86-Q386, H60-W324, L166-W324); (E) illustrates the recovered complex in which phosphatase folds as well as a the binding map are stabilized. The correlation map and flexibility profile acquired from the last 20 ns of this trajectory can be found in the main article, Figures 6(B) and 7, respectively.
(TIFF)

Figure S5 Snapshots from MD simulation of closed lid, ABA-bound PYR1 and HAB1, initially shifted away from each other by 15 Å, at 310°K: Immediately after minimizations and equilibrations (A), and after production runs of 2 ns (B), 8 ns (C), 23 ns (D) and 36 ns (E). In (A), the distance from PYR1 to HAB1 is already decreased to approximately 10 Å; in (B), PYR1 gate and loop Lα3β2 formed bonds with HAB1 (G86-R389, H60-W324) accompanied by a detachment of phosphatase residues P366-P411; in (C) more bonds between helix α5 and HAB1 are formed, stabilizing the complex; in (D) phosphatase has slightly rotated against PYR1, while the contacts remain stable; in (E) HAB1 folds are recovered and the complex acquires a structure similar to the crystallographic model.
(TIFF)

Figure S6 Snapshots of MD simulation for closed lid, ABA extracted PYR1 and HAB1 shifted away from each other by 15 Å, at 310 K: immediately after minimizations and equilibrations (A), and after production run of 1 ns (B), 5 ns (C), 9 ns (D), 15 ns (E), and 28 ns (F). In (A), the distance between S85 (the PYR1 gate) and F388 (HAB1) decreased to 8 A; in (B) the gate approached F388 forming unstable bond; in (C) phosphatase has rotated and its helix (containing residue 373) docked to the binding surface between PYR1's gate and α5; in (D) PYR1 helix α5 formed a bond with HAB1's helix; in (E) a distance between these helices increases; in (F) PYR1 has rotated slightly and new bonds between other residues of the same helices are formed. During the following 12 ns of simulation, the recovered complex remained stable. Binding surface is somehow displaced from the surface of complex PYR1-ABA-HAB1. The correlation map and flexibility profile acquired from the last 20 ns of this trajectory can be found in the main article, Figures 6(C) and 7, respectively.
(TIFF)

Figure S7 Snapshots of MD simulation for closed lid, ABA extracted PYR1 and HAB1 shifted from each other by 15 Å, at 310 K, from a different trajectory than in Figure S4. Immediately after equilibration (A), and after production run of 5 ns (B), 12 ns (C), 16 ns (D), 17 ns (E), and 20 ns (F). In (A), distance between PYR1 and HAB1 is 14 Å; in (B) the distance did not decrease yet, but PYR1 rotated; in (C) a bond developed between the gate and HAB1 β-strand (S85-D313); in (D) PYR1 is rotated again so that β2 (K63) is bound to HAB; in (E) HAB1 helices approach PYR1 helices α2 and α5, forming a bond; in (F) HAB1 rotates, forming more bonds with the receptor. Also, the phosphatase folds, becoming more compact. The recovered binding interface is different from that in PYR1-ABA-HAB1 complex.
(TIFF)

Figure S8 Basal Activity of apo-PYR1 Titrated against ABI2. Increasing amounts of PYR1 were titrated against a fixed amount of ABI2 (0.5 µM). The ratio of PYR1 : ABI2 is shown below each bar. The black bar represents the activity of ABI2 alone. The grey bars show the ABI2 activity observed for various combinations of PYR1 and ABI2. The red bar represents the activity of an equimolar concentration of PYR1 and ABI1 in the presence of 100 µM (+)-ABA. Each bar represents an average of three replicates and the standard deviations are indicated at the top of each bar. All protein was prepared fresh and used immediately. Details of protein preparation and assay are as described previously [6]. Essentially, the concentration of ABI2 was fixed at 0.5 uM and increasing concentrations of RCAR11 were added to the phosphatase in a 100 µl reaction mixture, in a buffer containing 100 mM Tris pH 7.9, 100 mM NaCl, 0.3 mM MnCl_2_ and 4 mM DTT. This mixture was pre- incubated for 15 min at 30°C and 1 mM substrate (1 mM 4-Methylumbelliferyl phosphate) was added to the reaction mixture which was further incubated for 1 hour at 30°C. Phosphatase activity was determined by spectrofluorometric analysis with the excitation wavelength was 355 nm and the emission wavelength at 460 nm.
(TIFF)

Figure S9 Effect of freeze-thaw cycles on apo- and holo- PYL5 activity. The constitutive inhibitory activity of PYL5 against ABI2 was tested after the proteins were subjected to different treatments. The PP2C activities of 0.4 µM ABI2 alone (black bars), ABI2 + 2.4 µM PYL5 (grey bars) and ABI2 + PYL5 + 10 µM (+)-ABA (red bars) are shown. The data sets represent (1) PP2C activities of freshly purified proteins, (2) fresh proteins with 10% glycerol, (3) proteins subject to one freeze-thaw cycle, (4) two freeze-thaw cycles (frozen for 4 days) and (5) three freeze-thaw cycles. Each bar represents an average of three replicates and the standard deviations are indicated on top of each bar. Protein was prepared and assayed as described in [6].
(TIFF)

Figure S10 Comparison of main chain flexibility profiles for PYR1 dimers: apo (ligand free) dimer (red line), 2ABA-bound dimer (green line), 1ABA-bound dimer (blue line) and pyrabactin-bound dimer (yellow line). Dashed lines indicate the regions of dimer binding. The average flexibility for the pyrabactin-bound dimer (yellow line) was calculated for the construct with mutation P88S. In 1ABA-bound dimer, the profile for ABA-containing chain is shown.
(TIFF)

Figure S11 RMS deviations of backbone atoms in core regions of our model structure for one-ABA-bound PYR1 dimer (prepared by modifying the starting structure 3NJO) against the crystallographic model of one-ABA-bound PYR1 dimer (3K3K) during last stages of NVT equilibration (400 ps–750 ps) and NPT equilibration (750 ps–950 ps). Simulations in the interval 0–400 ps (not shown) comprised 6 heating steps of 50 ps each followed by the initial 100 ps NVT equilibration of the modified 3NJO structure.
(TIFF)

Figure S12 The structural alignment of core regions of our model structure for one-ABA-bound PYR1 dimer (prepared by modifying the starting structure 3NJO) with the crystallographic model of one-ABA-bound-PYR1 dimer (3K3K) after completion of the NPT equilibration of the modified 3NJO structure.
(TIFF)

Table S1 Nearest inter-molecular neighbors in complexes of PYR/PYL ABA receptors with HAB1. Pink color represents receptor's residues, white color represents neighbor HAB1 residues, and green color represents neighbor ABA molecules.
(PDF)

## References

[1] Leung J, Giraudat J (1998) Abscisic acid signal transduction. Annu Rev Plant Physiol Plant Mol Biol 49: 199–222. 15012233 10.1146/annurev.arplant.49.1.199

[2] Nambara E, Marion-Poll A (2005) Abscisic acid biosynthesis and catabolism. Annu Rev Plant Biol 56: 165–185. 15862093 10.1146/annurev.arplant.56.032604.144046

[3] Cutler SR, Rodriguez PL, Finkelstein RR, Abrams SR (2010) Abscisic acid: emergence of a core signaling network. Annu Rev Plant Biol 61: 651–679. 20192755 10.1146/annurev-arplant-042809-112122

[4] Hirayama T, Shinozaki K (2007) Perception and transduction of abscisic acid signals: keys to the function of the versatile plant hormone ABA. Trends Plant Sci 12: 343–351. 17629540 10.1016/j.tplants.2007.06.013

[5] Klingler JP, Batelli G, Zhu JK (2010) ABA receptors: the START of a new paradigm in phytohormone signaling. J Exp Bot 61: 3199–3210. 20522527 10.1093/jxb/erq151 PMC3107536

[6] Ma Y, Szostkiewicz I, Korte A, Moes D, Yang Y, et al (2009) Regulators of PP2C phosphatase activity function as abscisic acid sensors. Science 324: 1064–1068. 19407143 10.1126/science.1172408

[7] Park SY, Fung P, Nishimura N, Jensen DR, Fuji H, et al (2009) Abscisic acid inhibits type 2C protein phosphatases via the PYR/PYL family of START proteins. Science 324: 1068–1071. 19407142 10.1126/science.1173041 PMC2827199

[8] Santiago J, Dupeux F, Round A, Antoni R, Park SY, et al (2009) The abscisic acid receptor PYR1 in complex with abscisic acid. Nature 462: 665–668. 19898494 10.1038/nature08591

[9] Nishimura N, Hitomi K, Arvai AS, Rambo RP, Hitomi C, et al (2009) Structural mechanism of abscisic acid binding and signaling by dimeric PYR1. Science 326: 1373–1379. 19933100 10.1126/science.1181829 PMC2835493

[10] Peterson FC, Burgie ES, Park SY, Jensen DR, Weiner JJ, et al (2010) Structural basis for selective activation of ABA receptors. Nat Struct Mol Biol 17: 1109–1113. 20729860 10.1038/nsmb.1898 PMC2933299

[11] Melcher K, Ng LM, Zhou XE, Soon FF, Xu Y, et al (2009) A gate-latch-lock mechanism for hormone signaling by abscisic acid receptors. Nature 462: 602–608. 19898420 10.1038/nature08613 PMC2810868

[12] Miyazono K, Miyukawa T, Sawano Y, Kubota K, Kang HJ, et al (2009) Structural basis of abscisic acid signaling. Nature 462: 609–614. 19855379 10.1038/nature08583

[13] Hubbard KE, Nishimura N, Hitomi K, Getzoff ED, Schroeder JI (2010) Early abscisic acid signal transduction mechanisms: newly discovered components and newly emerging questions. Genes & Dev 24: 1695–1708. 20713515 10.1101/gad.1953910 PMC2922499

[14] Melcher K, Xu Y, Ng LM, Zhou XE, Soon FF, et al (2010) Identification and mechanism of ABA receptor. Nat Struct Mol Biol 17: 1102–1108. 20729862 10.1038/nsmb.1887 PMC2933329

[15] Hao Q, Yin P, Li W, Wang L, Yan C, et al (2011) The molecular basis of ABA-independent inhibition of PP2C by a subclass of PYL proteins. Mol Cell 42: 662–672. 21658606 10.1016/j.molcel.2011.05.011

[16] Dupeux F, Santiago J, Betz K, Twycross J, Park SY, et al (2011) A thermodynamic switch modulates abscisic acid receptor sensitivity. EMBO J 30: 4171–4184. 21847091 10.1038/emboj.2011.294 PMC3199383

[17] Dupeux F, Antoni R, Betz K, Santiago J, Gonzalez-Guzman M, et al (2011) Modulation of abscisic acid signaling in vivo by an engineered receptor-insensitive protein phosphatase type 2C allele. Plant Physiol 156: 106–116. 21357183 10.1104/pp.110.170894 PMC3091035

[18] Mosquna A, Peterson FC, Park SY, Lozano-Juste J, Volkman BF, et al (2012) Potent and selective activation of abscisic acid receptors in vivo by mutational stabilization of their agonist-bound conformation. Proc Nat Acad Sci USA 108: 20838–43. 10.1073/pnas.1112838108 PMC3251050 22139369

[19] Wieligmann K, Pineda De Castro LF, Zacharias M (2002) Molecular dynamics simulations of the free and complexed N-terminal SH2 domain of SHP-2. In Silico Biol 2: 305–311. 12542415

[20] Peters GH, Frimurer TM, Andersen JN, Olsen OH (2000) Molecular dynamics simulations of protein-tyrosine phosphatase 1B. II. Substrate-enzyme interactions and dynamics. Biophys J 78: 2191–2200. 10777720 10.1016/S0006-3495(00)76768-3 PMC1300813

[21] Ashokan K (2010) Docking studies on abscisic acid receptor pyrabactin receptor 1 (PYR1) and pyrabactin-like receptor (PYL1). Int J Environm Studies 1: 314–322.

[22] Noguti T, Go N (1985) Efficient Monte Carlo method for simulation of fluctuating conformations of native protein. Biopolymers 24: 527–546. 3986295 10.1002/bip.360240308

[23] Garcia A (1992) Large-amplitude nonlinear motions in proteins. Phys Rev Lett 68: 2696–2699. 10045464 10.1103/PhysRevLett.68.2696

[24] Amadei A, Linssen AB, Berendsen HJ (1993) Essential dynamics of proteins. Proteins Struct Funct Genetics 17: 412–425. 10.1002/prot.340170408 8108382

[25] Emberly EG, Mukhopadhyay R, Wingreen NS, Tang C (2003) Flexibility of alpha-helices: Results on a statistical analysis of database protein structures. J Mol Biol 327: 229–237. 12614621 10.1016/s0022-2836(03)00097-4

[26] Tozzini V (2005) Coarse-grained models for proteins. Curr Opin Struct Biol 15: 144–150. 15837171 10.1016/j.sbi.2005.02.005

[27] Yesylevskyy SO, Kharkyanen VN, Demchenko AP (2006) Dynamic protein domains: identification, interdependence, and stability. Biophys J 91: 670–685. 16632509 10.1529/biophysj.105.078584 PMC1483087

[28] Sakuraba S, Joti Y, Kitao A (2010) Detecting coupled collective motions in protein by independent subspace analysis. J Chem Phys 133: 185102. 21073231 10.1063/1.3498745

[29] Zhuravlev PI, Papoian GA (2010) Protein functional landscapes, dynamics, allostery: a tortuous path towards a universal theoretical framework. Q Rev Biophys 43: 295–332. 20819242 10.1017/S0033583510000119

[30] Pearlman DA, Case DA, Caldwell JW, Ross WS, Cheatam III TE, et al (1995) AMBER, a package of computer programs for applying molecular mechanics, normal mode analysis, molecular dynamics and free energy calculations to simulate the structural and energetic properties of molecules. Comp Phys Comm 91: 1–41.

[31] Stepanova M (2007) Dynamics of essential collective motions in proteins: Theory. Phys Rev E 76: 051918–15. 10.1103/PhysRevE.76.051918 18233698

[32] Blinov N, Berjanskii M, Wishart DS, Stepanova M (2009) Structural domains and main-chain flexibility in prion proteins. Biochemistry 48: 1488–1497. 19178154 10.1021/bi802043h

[33] Potapov A, Stepanova M (2012) Conformational modes in biomolecules: Dynamics and approximate invariance. Phys Rev E 85: 020901–4. 10.1103/PhysRevE.85.020901 22463145

[34] Issack BB, Berjanskii M, Wishart DS, Stepanova M (2012) Exploring the essential collective dynamics of interacting proteins: Application to prion protein dimers. Proteins Str Funct Bioinorm 80: 1847–65. 10.1002/prot.24082 22488640

[35] Santo KP, Berjanskii M, Wishart DS, Stepanova M (2011) Comparative analysis of essential collective dynamics and NMR-derived flexibility profiles in evolutionary diverse prion proteins. Prion 5: 188–200. 21869604 10.4161/pri.5.3.16097 PMC3226046

[36] Bernstein FC, Koetzle TF, Williams GJ, Meyer EF, Brice Jr MD, et al (1977) The Protein Data Bank: a computer-based archival file for macromolecular structures. J Mol Biol 112: 535–542. 875032 10.1016/s0022-2836(77)80200-3

[37] Halle B (2002) Flexibility and packing in proteins. Proc Nat Acad Sci USA 99: 1274–1279. 11818549 10.1073/pnas.032522499 PMC122180

[38] Zhang X, Zhang Q, Xin Q, Yu L, Wang Z, et al (2012) Complex structures of the abscisic acid receptor PYL3/RCAR13 reveal a unique regulatory mechanism. Structure 20: 780–790. 22579247 10.1016/j.str.2012.02.019

[39] Antoni R, Gonzalez-Guzman M, Rodriguez L, Rodriguez A, Pizzio GA, et al (2012) Selective inhibition of clade a phosphatases type 2C by PYR/PYL/RCAR abscisic acid receptors. Plant Physiol 158: 970–980. 22198272 10.1104/pp.111.188623 PMC3271782

[40] Accelrys Discovery Studio, version 3.0.0.10321. Available: http://accelrys.com/. Accessed May 26, 2011.

[41] Sousa Da Silva AW, Vranken WF (2012) ACPYPE - AnteChamber PYthon Parser interface. BMC Res Notes 5: 1–8. 22824207 10.1186/1756-0500-5-367 PMC3461484

[42] Ribeiro A, Horta B, de Alencastro R (2008) MKTOP: A program for automatic construction of molecular topologies. J Braz Chem Soc 19: 1433–1435.

[43] Berendsen HJC, van der Spoel D, van Drunen R (1995) GROMACS - A message-passing parallel molecular dynamics implementation. Comp Phys Comm 91: 43–56.

[44] Case DA, Darden TA, Cheatham III TE, Simmerling CL, Wang J, et al.. (2010) AMBER 11. San Francisco: University of California.

[45] Berendsen HJC, Postma JPM, van Gunsteren WF, Dinola A, Haak JR (1984) Molecular dynamics with coupling to an external bath. J Chem Phys 81: 3684–3690.

[46] Humphrey W, Dalke A, Schulten K (1996) VMD: visual molecular dynamics. J Mol Graph 14: 33–8, 27–8. 8744570 10.1016/0263-7855(96)00018-5

[47] Barakat K, Issack BB, Stepanova M, Tuszynski J (2011) Effects of temperature on the p53-DNA binding interactions and their dynamical behavior: comparing the wild type to the R248Q mutant. PLoS One 6: e27651. 22110706 10.1371/journal.pone.0027651 PMC3218007

