# Supplementary figures and images for: Molecular Mechanisms in the Activation of Abscisic Acid Receptor PYR1
Source: PLoS Comput Biol. 2013 Jun 27;9(6):e1003114. doi: 10.1371/journal.pcbi.1003114 (PMC3694813; doi:10.1371/journal.pcbi.1003114)

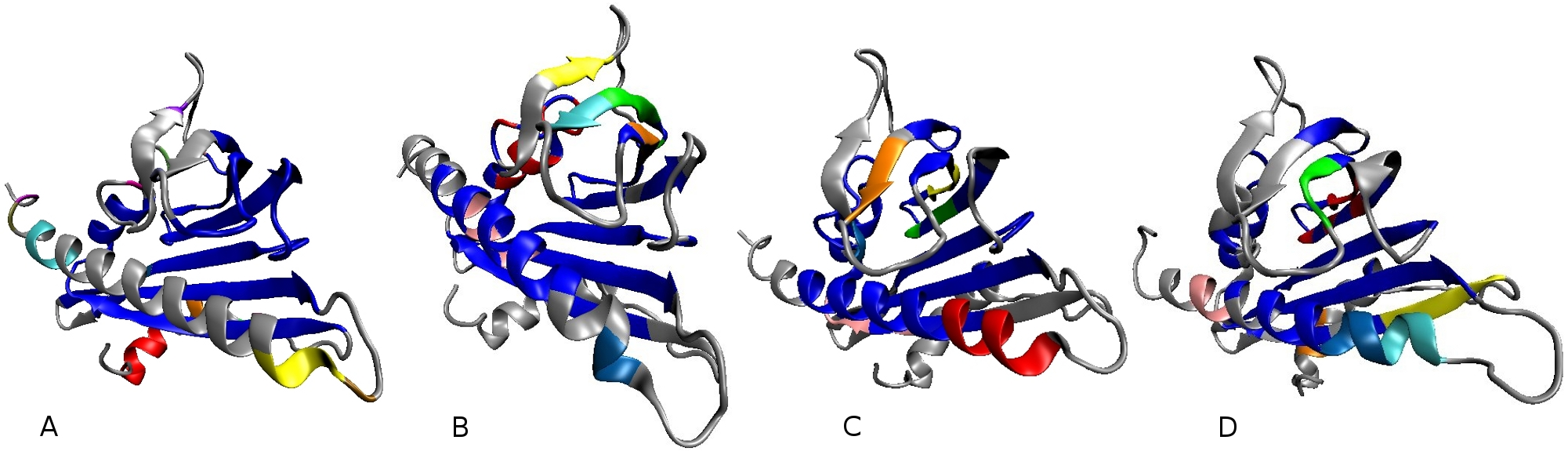

Supplement: Figure S1 — Dynamical domains of correlated motion for the pyrabactin receptor (PYR1), closed lid and ABA-extracted. Simulations were performed at (A) 281 K, (B) 300 K, (C) 310 K, (D) 325 K. Interdomain distance d = 0.0015 has been adopted. The color scheme is as in Figure 4. The largest domain, coloured blue, indicates the most extensive dynamical correlations. (TIFF) [file pcbi.1003114.s001.tiff]

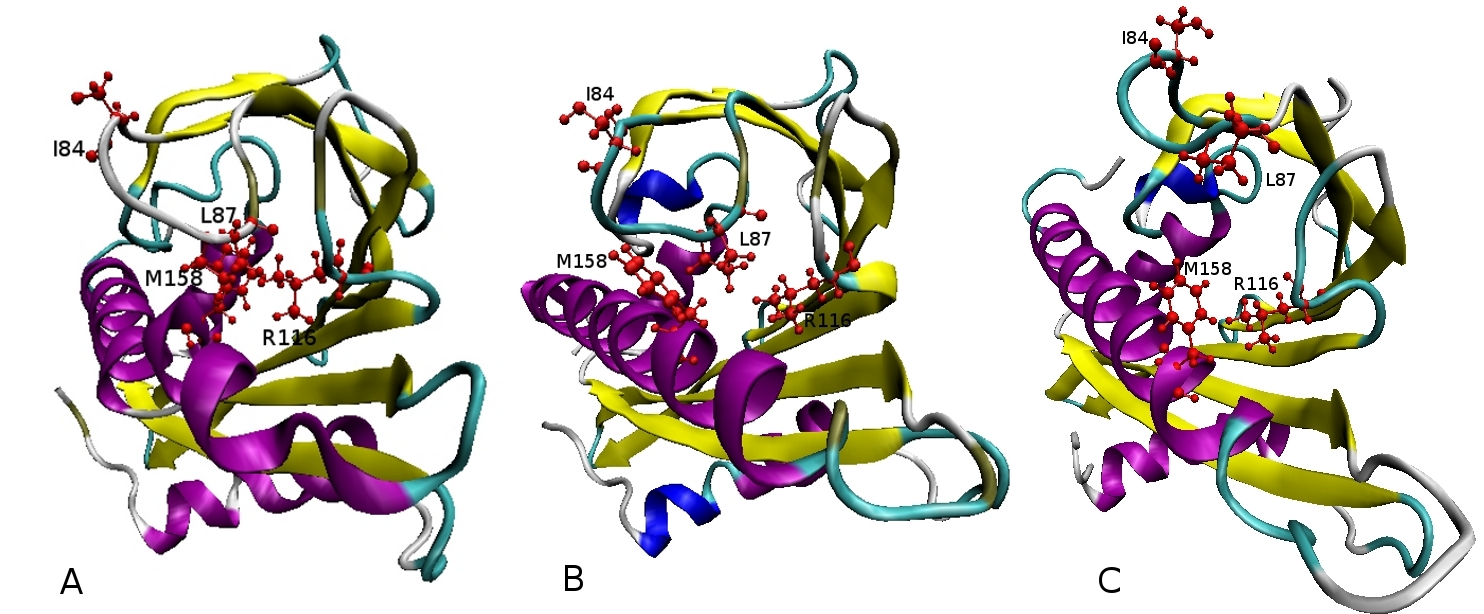

Supplement: Figure S2 — PYR1 closed lid, ABA extracted construct after 20 ns of simulations at 300 K (A), 310 K (B), and 325 K (C). In (A) structure retains the closed lid conformation, in (B) the gate and latch have decoupled, however the contact of L87 with M158 is observed in approximately 30% of simulation snapshots; in (C) the gate detached from the latch and helix α5. (TIFF) [file pcbi.1003114.s002.tiff]

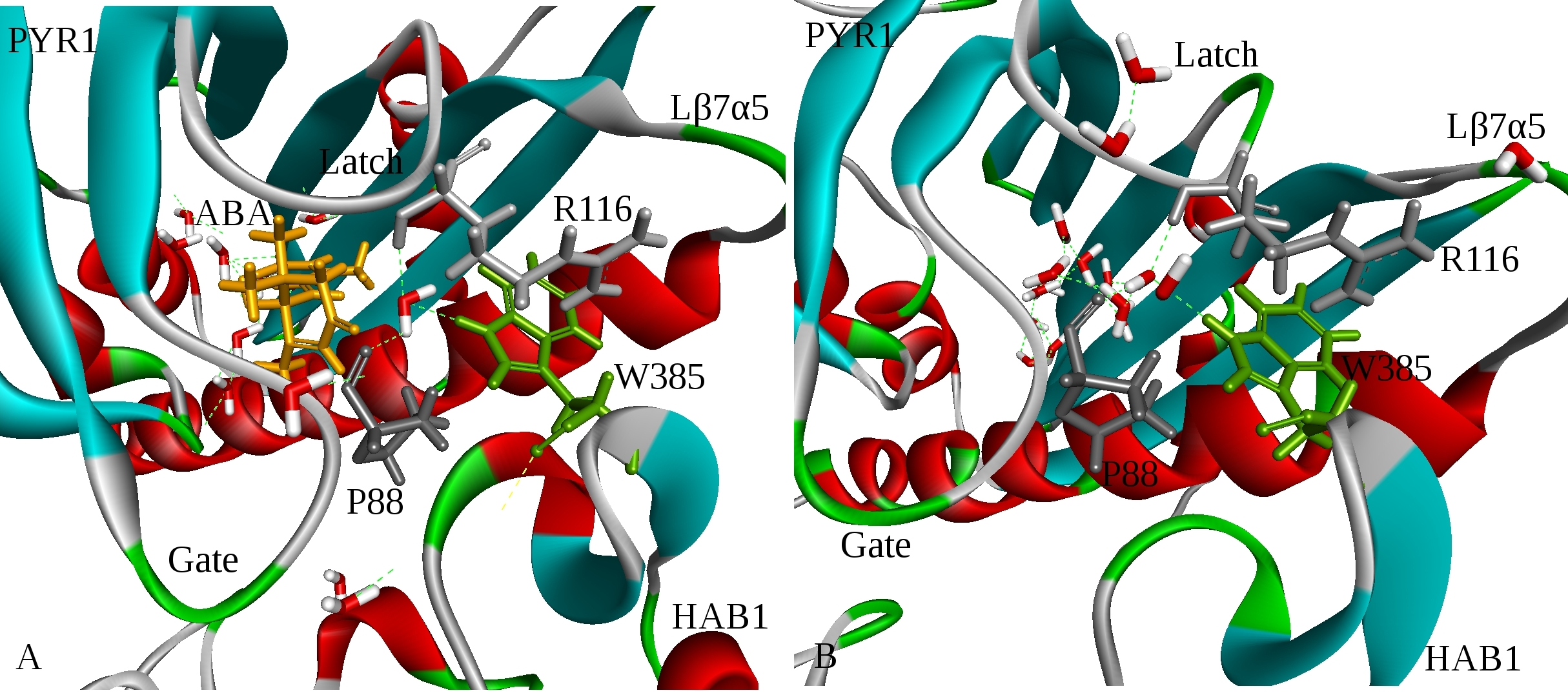

Supplement: Figure S3 — Close up of the binding area for ABA-bound (A) and ABA-free (B) PYR1-HAB1 complexes obtained from MD simulations using Accelrys VS. ABA and the residues W385, R116 and P88 are shown in orange, green, light grey and dark grey sticks, respectively. The water molecules are shown as red-and-white sticks. (TIFF) [file pcbi.1003114.s003.tiff]

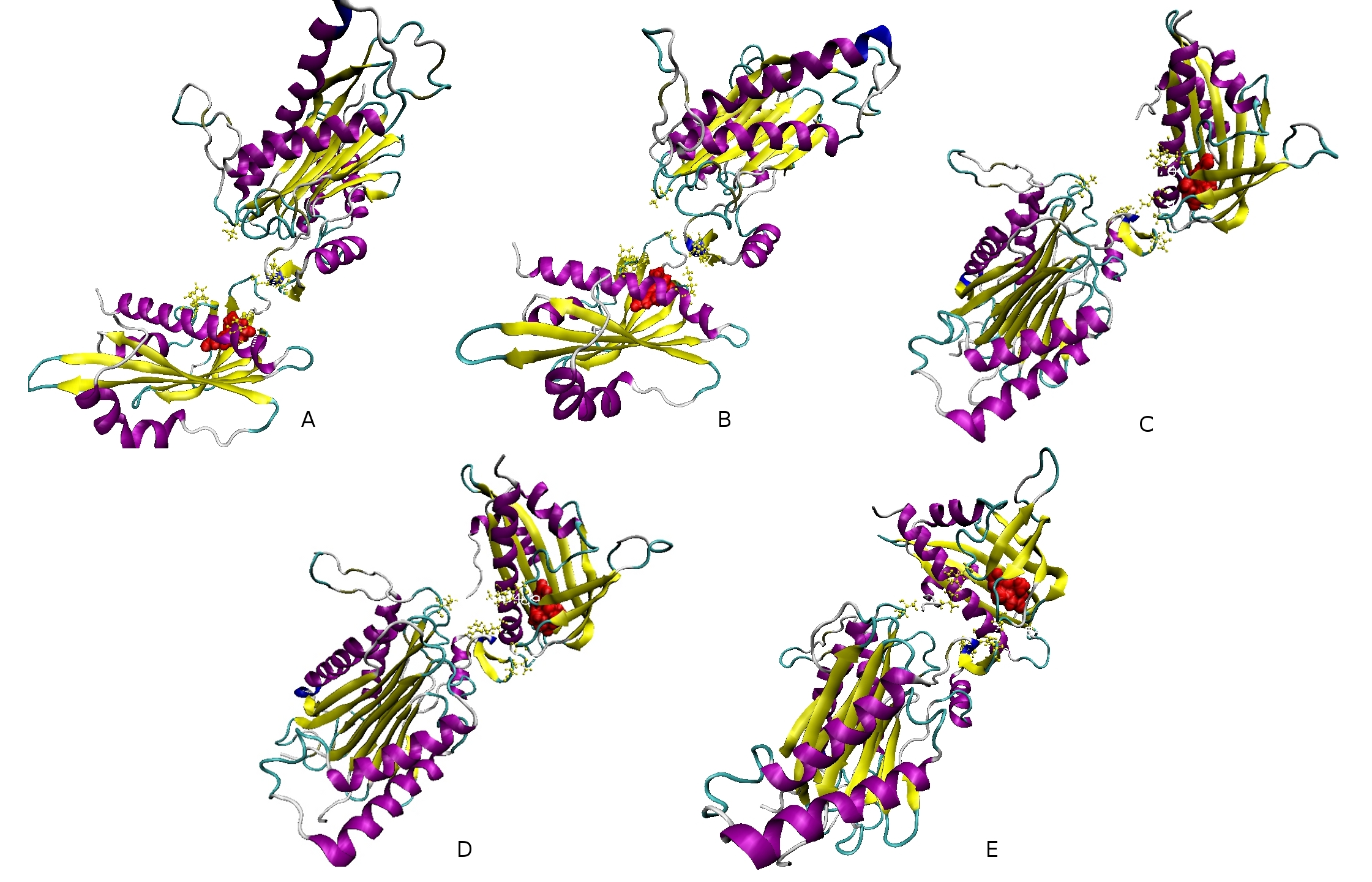

Supplement: Figure S4 — Snapshots from MD simulation of closed lid, ABA-bound PYR1 and HAB1, initially shifted away from each other by 15 Å, at 310°K (different trajectory than in Figure S2): Immediately after minimizations and equilibrations (A), and after production run of 4 ns (B), 13 ns (C), 18 ns (D), and 20 ns. In (A), the distance from PYR1 to HAB1 is decreased to 5 Å; in (B), binding of HAB1 to helix α5 of PYR1 (M158-F391) occurred; (C) captures HAB1 slowly approaching the rest of binding surface; in (D), more bonds are formed (G86-Q386, H60-W324, L166-W324); (E) illustrates the recovered complex in which phosphatase folds as well as a the binding map are stabilized. The correlation map and flexibility profile acquired from the last 20 ns of this trajectory can be found in the main article, Figures 6(B) and 7, respectively. (TIFF) [file pcbi.1003114.s004.tiff]

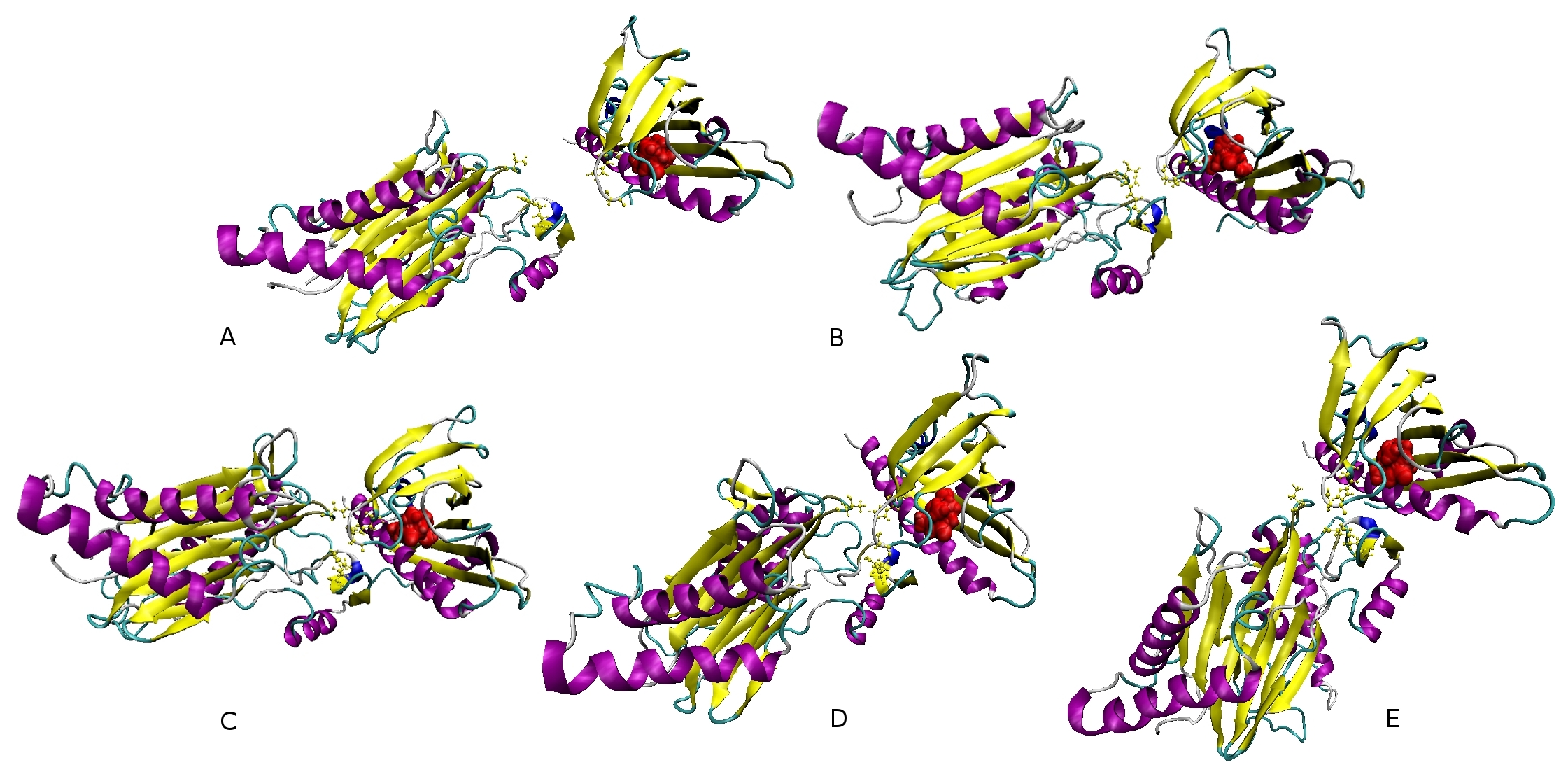

Supplement: Figure S5 — Snapshots from MD simulation of closed lid, ABA-bound PYR1 and HAB1, initially shifted away from each other by 15 Å, at 310°K: Immediately after minimizations and equilibrations (A), and after production runs of 2 ns (B), 8 ns (C), 23 ns (D) and 36 ns (E). In (A), the distance from PYR1 to HAB1 is already decreased to approximately 10 Å; in (B), PYR1 gate and loop Lα3β2 formed bonds with HAB1 (G86-R389, H60-W324) accompanied by a detachment of phosphatase residues P366-P411; in (C) more bonds between helix α5 and HAB1 are formed, stabilizing the complex; in (D) phosphatase has slightly rotated against PYR1, while the contacts remain stable; in (E) HAB1 folds are recovered and the complex acquires a structure similar to the crystallographic model. (TIFF) [file pcbi.1003114.s005.tiff]

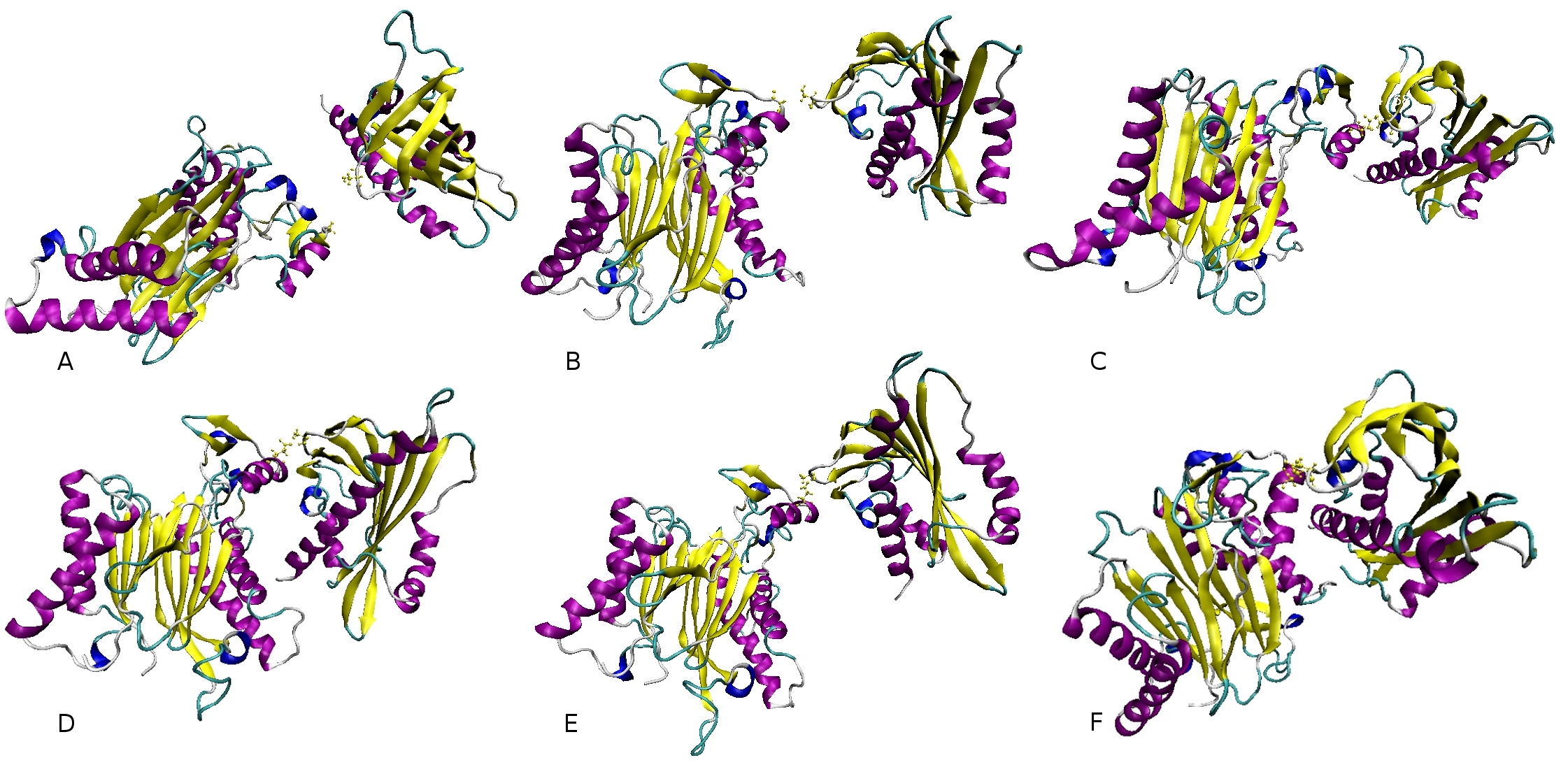

Supplement: Figure S6 — Snapshots of MD simulation for closed lid, ABA extracted PYR1 and HAB1 shifted away from each other by 15 Å, at 310 K: immediately after minimizations and equilibrations (A), and after production run of 1 ns (B), 5 ns (C), 9 ns (D), 15 ns (E), and 28 ns (F). In (A), the distance between S85 (the PYR1 gate) and F388 (HAB1) decreased to 8 A; in (B) the gate approached F388 forming unstable bond; in (C) phosphatase has rotated and its helix (containing residue 373) docked to the binding surface between PYR1's gate and α5; in (D) PYR1 helix α5 formed a bond with HAB1's helix; in (E) a distance between these helices increases; in (F) PYR1 has rotated slightly and new bonds between other residues of the same helices are formed. During the following 12 ns of simulation, the recovered complex remained stable. Binding surface is somehow displaced from the surface of complex PYR1-ABA-HAB1. The correlation map and flexibility profile acquired from the last 20 ns of this trajectory can be found in the main article, Figures 6(C) and 7, respectively. (TIFF) [file pcbi.1003114.s006.tiff]

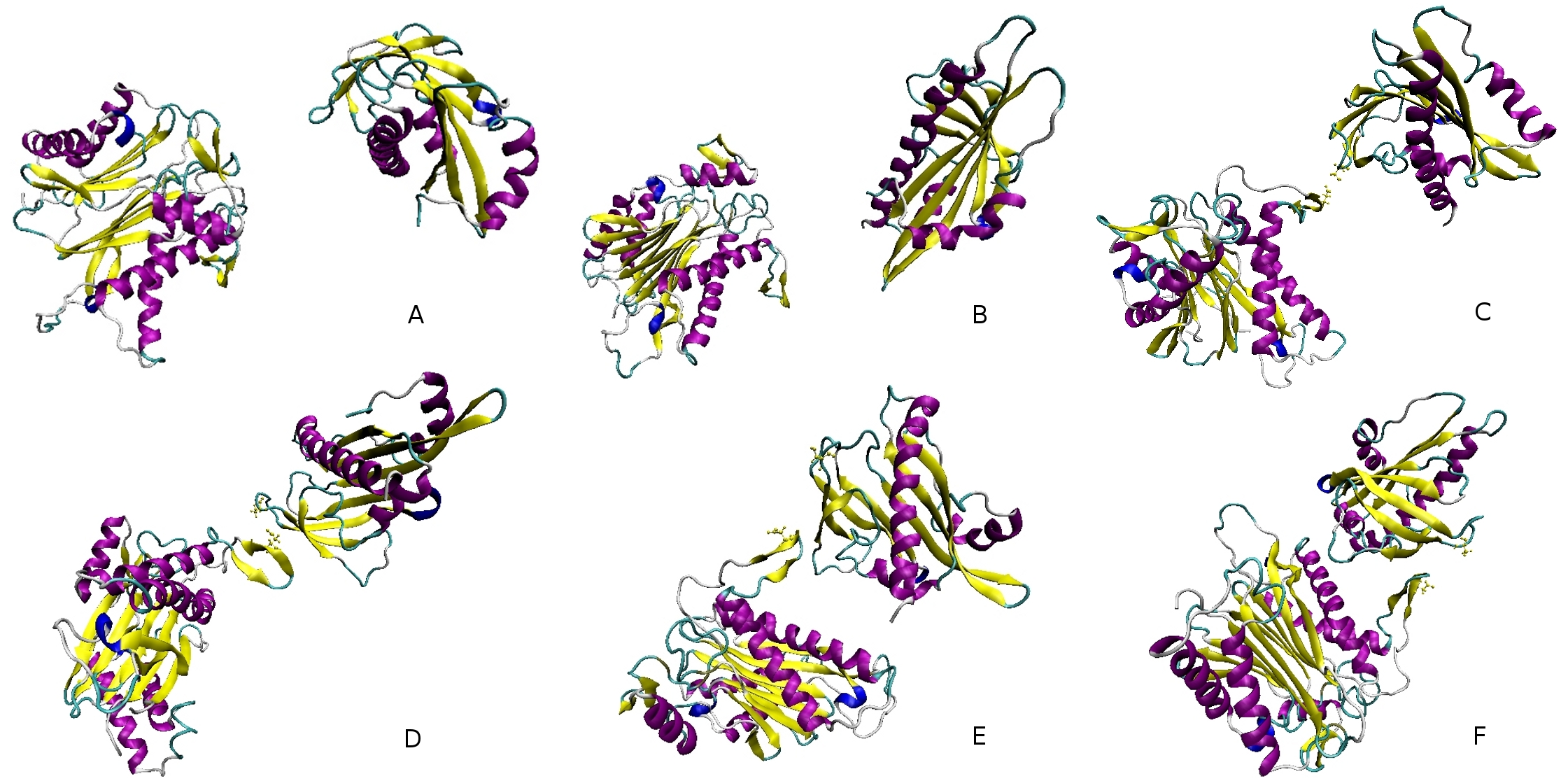

Supplement: Figure S7 — Snapshots of MD simulation for closed lid, ABA extracted PYR1 and HAB1 shifted from each other by 15 Å, at 310 K, from a different trajectory than in Figure S4. Immediately after equilibration (A), and after production run of 5 ns (B), 12 ns (C), 16 ns (D), 17 ns (E), and 20 ns (F). In (A), distance between PYR1 and HAB1 is 14 Å; in (B) the distance did not decrease yet, but PYR1 rotated; in (C) a bond developed between the gate and HAB1 β-strand (S85-D313); in (D) PYR1 is rotated again so that β2 (K63) is bound to HAB; in (E) HAB1 helices approach PYR1 helices α2 and α5, forming a bond; in (F) HAB1 rotates, forming more bonds with the receptor. Also, the phosphatase folds, becoming more compact. The recovered binding interface is different from that in PYR1-ABA-HAB1 complex. (TIFF) [file pcbi.1003114.s007.tiff]

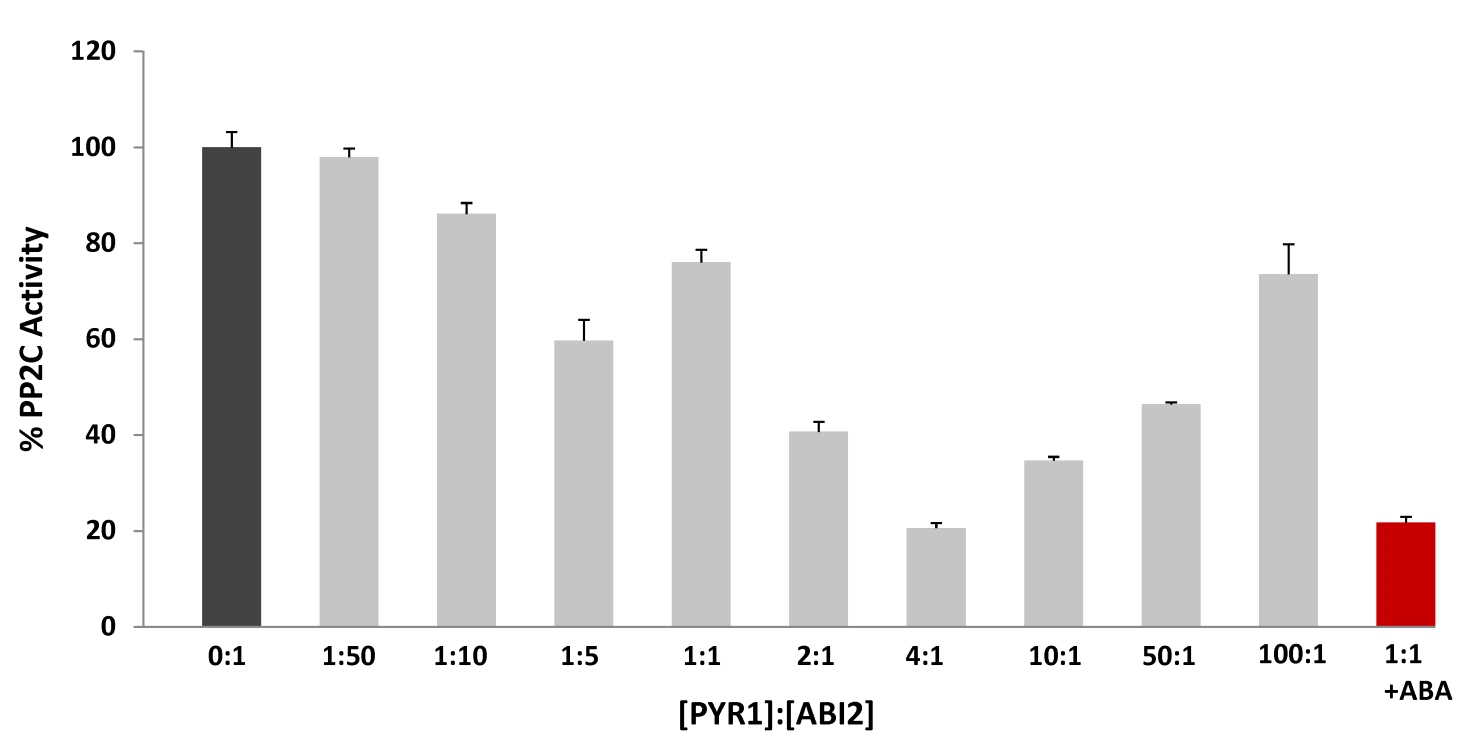

Supplement: Figure S8 — Basal Activity of apo-PYR1 Titrated against ABI2. Increasing amounts of PYR1 were titrated against a fixed amount of ABI2 (0.5 µM). The ratio of PYR1 : ABI2 is shown below each bar. The black bar represents the activity of ABI2 alone. The grey bars show the ABI2 activity observed for various combinations of PYR1 and ABI2. The red bar represents the activity of an equimolar concentration of PYR1 and ABI1 in the presence of 100 µM (+)-ABA. Each bar represents an average of three replicates and the standard deviations are indicated at the top of each bar. All protein was prepared fresh and used immediately. Details of protein preparation and assay are as described previously [6]. Essentially, the concentration of ABI2 was fixed at 0.5 uM and increasing concentrations of RCAR11 were added to the phosphatase in a 100 µl reaction mixture, in a buffer containing 100 mM Tris pH 7.9, 100 mM NaCl, 0.3 mM MnCl2 and 4 mM DTT. This mixture was pre- incubated for 15 min at 30°C and 1 mM substrate (1 mM 4-Methylumbelliferyl phosphate) was added to the reaction mixture which was further incubated for 1 hour at 30°C. Phosphatase activity was determined by spectrofluorometric analysis with the excitation wavelength was 355 nm and the emission wavelength at 460 nm. (TIFF) [file pcbi.1003114.s008.tiff]

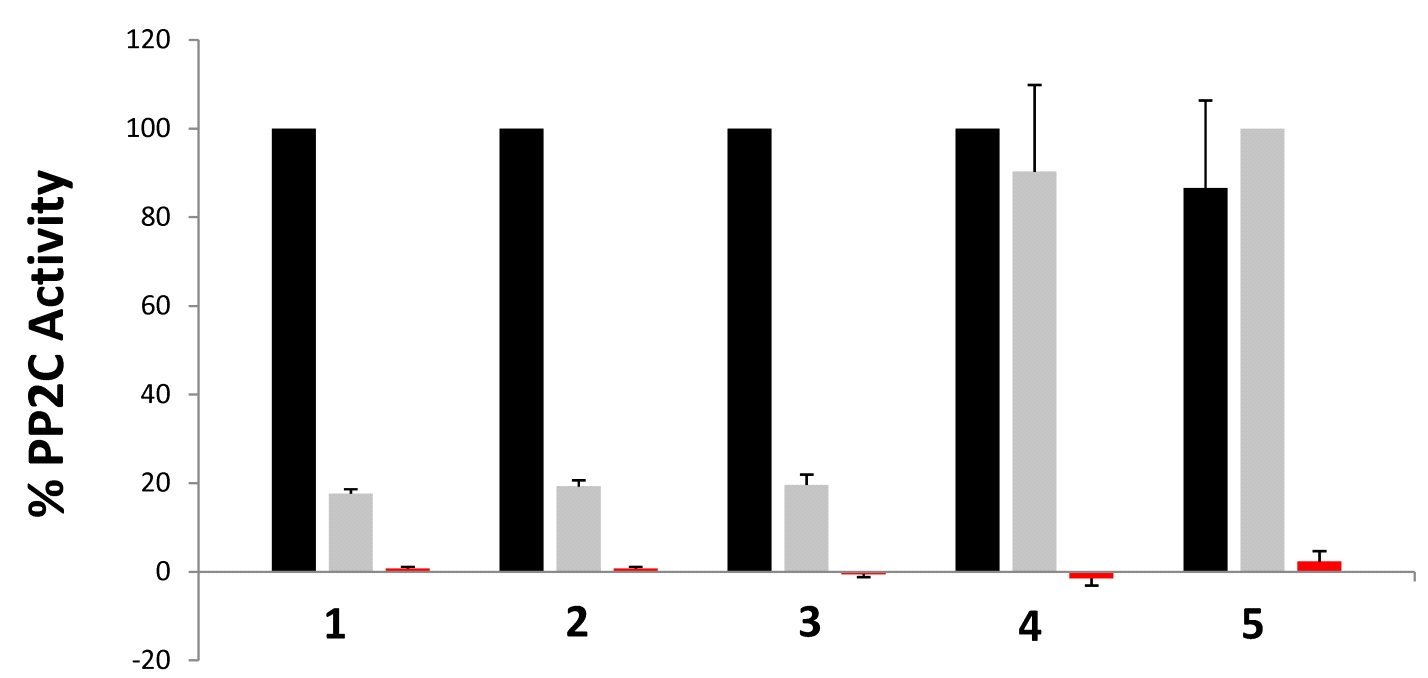

Supplement: Figure S9 — Effect of freeze-thaw cycles on apo- and holo- PYL5 activity. The constitutive inhibitory activity of PYL5 against ABI2 was tested after the proteins were subjected to different treatments. The PP2C activities of 0.4 µM ABI2 alone (black bars), ABI2 + 2.4 µM PYL5 (grey bars) and ABI2 + PYL5 + 10 µM (+)-ABA (red bars) are shown. The data sets represent (1) PP2C activities of freshly purified proteins, (2) fresh proteins with 10% glycerol, (3) proteins subject to one freeze-thaw cycle, (4) two freeze-thaw cycles (frozen for 4 days) and (5) three freeze-thaw cycles. Each bar represents an average of three replicates and the standard deviations are indicated on top of each bar. Protein was prepared and assayed as described in [6]. (TIFF) [file pcbi.1003114.s009.tiff]

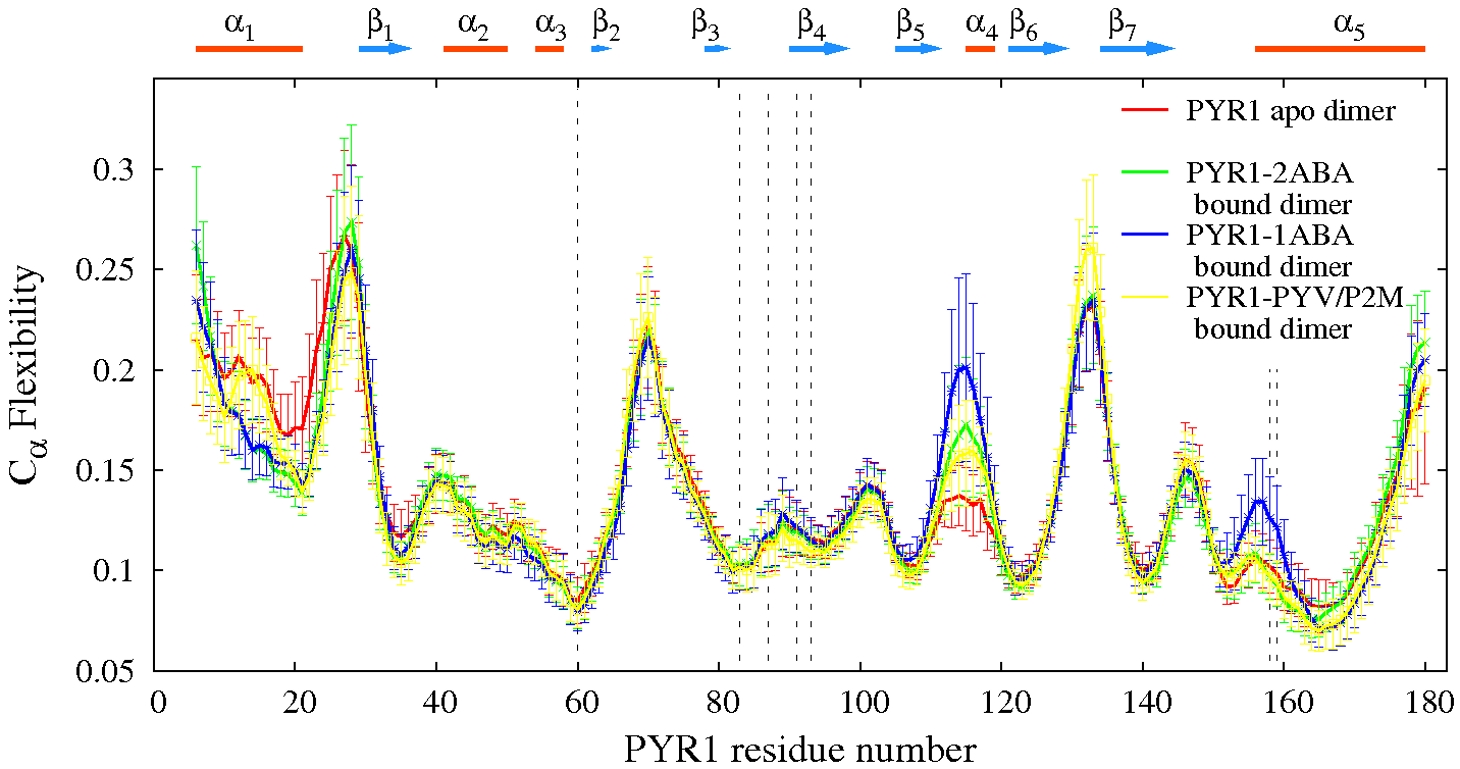

Supplement: Figure S10 — Comparison of main chain flexibility profiles for PYR1 dimers: apo (ligand free) dimer (red line), 2ABA-bound dimer (green line), 1ABA-bound dimer (blue line) and pyrabactin-bound dimer (yellow line). Dashed lines indicate the regions of dimer binding. The average flexibility for the pyrabactin-bound dimer (yellow line) was calculated for the construct with mutation P88S. In 1ABA-bound dimer, the profile for ABA-containing chain is shown. (TIFF) [file pcbi.1003114.s010.tiff]

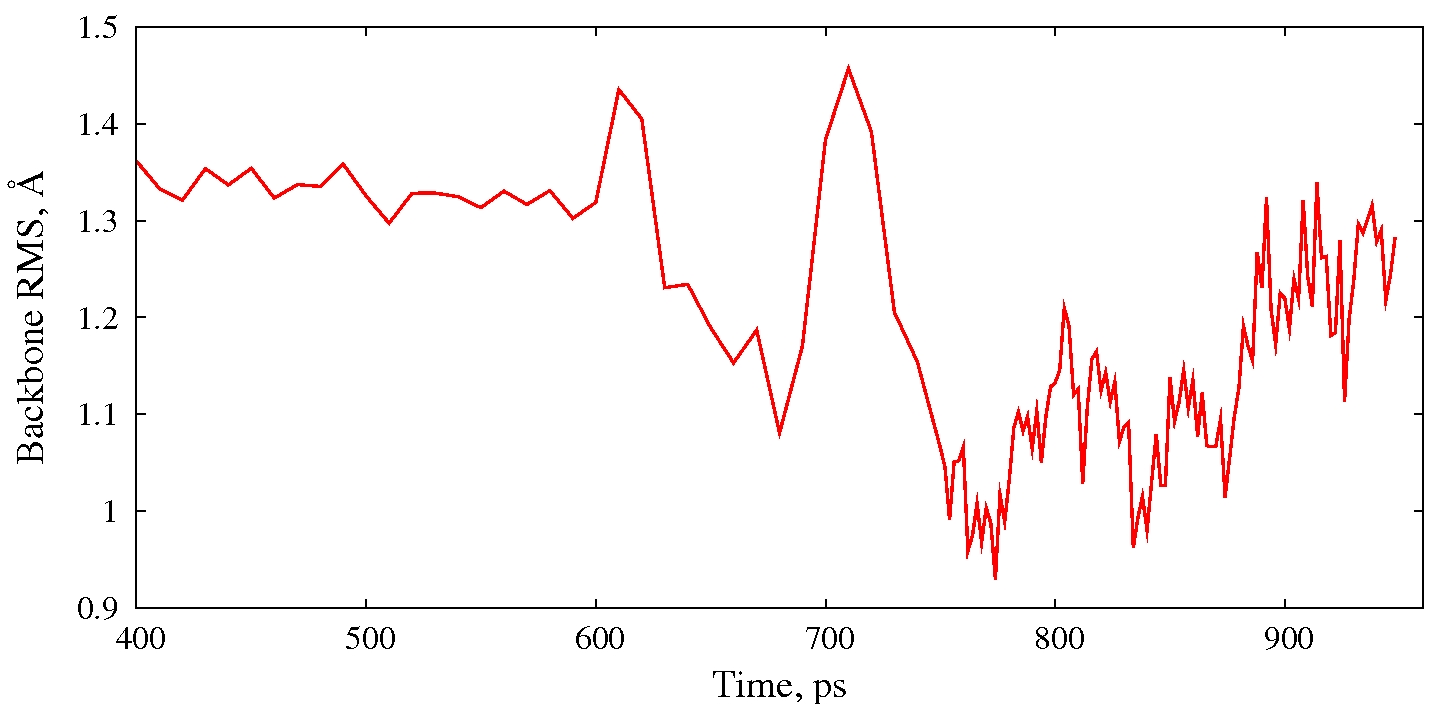

Supplement: Figure S11 — RMS deviations of backbone atoms in core regions of our model structure for one-ABA-bound PYR1 dimer (prepared by modifying the starting structure 3NJO) against the crystallographic model of one-ABA-bound PYR1 dimer (3K3K) during last stages of NVT equilibration (400 ps–750 ps) and NPT equilibration (750 ps–950 ps). Simulations in the interval 0–400 ps (not shown) comprised 6 heating steps of 50 ps each followed by the initial 100 ps NVT equilibration of the modified 3NJO structure. (TIFF) [file pcbi.1003114.s011.tiff]

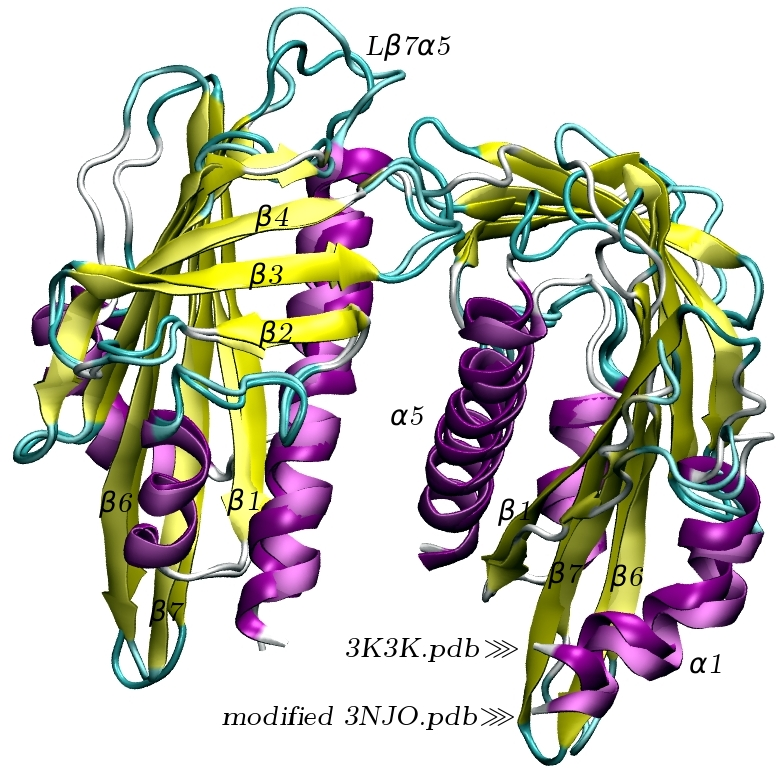

Supplement: Figure S12 — The structural alignment of core regions of our model structure for one-ABA-bound PYR1 dimer (prepared by modifying the starting structure 3NJO) with the crystallographic model of one-ABA-bound-PYR1 dimer (3K3K) after completion of the NPT equilibration of the modified 3NJO structure. (TIFF) [file pcbi.1003114.s012.tiff]
