# Supplementary material for: Molecular Mechanisms in the Activation of Abscisic Acid Receptor PYR1
Source: PLoS Comput Biol. 2013 Jun 27;9(6):e1003114. doi: 10.1371/journal.pcbi.1003114 (PMC3694813; doi:10.1371/journal.pcbi.1003114)
Supplement: Table S1 — Nearest inter-molecular neighbors in complexes of PYR/PYL ABA receptors with HAB1. Pink color represents receptor's residues, white color represents neighbor HAB1 residues, and green color represents neighbor ABA molecules. (PDF) [file pcbi.1003114.s013.pdf]

Nearest inter-molecular neighbors in complexes of PYR/PYL ABA receptors with HAB1.

Pink - receptor's residues; white - neighbor HAB1 residues; green - neighbor ABA molecules.

		Lα3β2			β2							Gate												Latch						Lβ7αs						Helix αs										
I		K59	H60	F61	I62	K63	S64	V81	I82	V83	I84	S85	G86	L87	P88	A89	N90	T91	S92	E94	F108	I110	G114	H115	R116	L117	Y120	S122	E141	P148	G150	N151	S152	D154	D155	T156	M158	F159	A160	T162	V163	V164	L166	N167	Q169	K170
PYR1-ABA-HAB1		ABA	S322	ABA		S200		ABA		ABA	G246	R199	H245	ABA	ABA	ABA		ABA	ABA	ABA	ABA	ABA	ABA	ABA	ABA	ABA	ABA	ABA	ABA	W385		I383			I383	W385	K381	ABA	ABA	F391	ABA	ABA	E323	ABA		
3QN1.pdb			T324	T324		E201					T324	E203	G246	Q386	W385	Q386									Q384	W385									W385		I383	I383		V393			T324			
[17]				V393								G244	R389	R389	Q386										W385							W385					F391	W385		Y404			Y404			
				Y404								H245	V393	V393	R389										Q386													F391		L405						
												G246	L394		G392																							G392								
												G247	A395		V393																							V393								
II	M1	K59		F61	I62			V81		V83	I84	S85	G86	L87	P88	A89	N90	T91	S92	E94	F108	I110	G114	H115	R116	L117	Y120	S122	E141				S152	D154	D155	T156	M158	F159	A160	T162	V163	V164	L166	N167	Q169	
PYR1-ABA-HAB1	E320	ABA		ABA	ABA			ABA		ABA	W382	W382	Q381	ABA	ABA	ABA	ABA	ABA	ABA	ABA	ABA	ABA	ABA	ABA	ABA	ABA	ABA	ABA	ABA				K378	G377	K378	G376	G376	ABA	ABA	W382	ABA	ABA	W382	ABA	E320	
3QN1.pdb	T321			W382									W382	W382	I380										I380									K378	I380		G377	I380		F388	W382	Y401	V390		T321	
shifted	R400			V390									Q383	Q383	Q381										Q381									I380			K378	Q381		V390			Y401			
& recovered	Y401												R386		W382																						I380	W382		Y401						
after 30 ns															Q383																						F388	F388		L402						
																																					Y405	G389								
III	H39	K59	H60	F61	I62	K63	S64				I84	S85																E132	N133	R134													L166	K170	E176	R180
PYR1-HAB1	K285	N374	D367	N374	N374	I372	G376				E373	Y369																N274	N274	N274													A370	R371	K285	D284
3QN1.pdb	E277		A370			E373					N374	E373																T275		G276														N374		K285
ABA-extracted			R371			N374																						G276		E277																T288
shifted			N374			A375																								G278																S289
& recovered						G376																								V281																
after 30 ns						G377																								Q282																
																														K285																
IV		K59	P60	F61		K63				V83	I84	S85	G86	L87	P88	A89		T91	S92	E94	F108	I110		H115	R116	L117	Y120		E141	P148		N151			D155	T156	M158	F159	A160	T162	V163	V164	L166	N167		K170
PYR1-ABA-HAB1		ABA	S322	ABA		S200				ABA	G246	R199	H245	ABA	ABA	ABA		ABA	ABA	ABA	ABA	ABA		ABA	Q384	ABA	ABA		ABA	W385		I383			K381	W385	K381	ABA	ABA	F391	ABA	ABA	E323	ABA		S322
3ZVU.pdb			E323	T324		E201					G247	E203	G246	Q386	W385	Q386									W385	W385						Q384			I383		I383	I383		V393	V393		W324			E323
mutant H60P			T324	V393							T324	G244	R389	R389	Q386										Q386							W385			W385		F391	W385		Y404			Y404			
[16]				Y404								H245	V393	V393	R389																						G392	F391		L405						
												G246	L394		G392																							G392								
												G247	A395		V393																							V393								
V			K64	H65	F66		K68	R69	V85	V87	I88	S89	G90	L91	P92	A93		T95	S96	E98	F112	V114		H119	R120	L121	Y124	S126	E147	P154		N157			D161	T162	M164	F165	V166	T168	V169	V170	L172	N173		K176
PYL2-ABA-HAB1			ABA	S322	ABA		S200	E201	ABA	ABA	G246	R199	R199	ABA	ABA	ABA		ABA	ABA	ABA	ABA	ABA		ABA	ABA	ABA	ABA	ABA	ABA	W385		I383			K381	W385	K381	ABA	ABA	F391	ABA	ABA	E323	ABA		E323
3KB3.pdb				E323	T324		E201				T324	E203	H245	Q386	W385	Q386									W385	W385						Q384			I383		I383	I383		V393	V393		Y404			
[11]				T324	V393							G244	G246	R389	Q386										Q386							W385			Q384		F391	W385		Y404						
					Y404							H245	R389	V393	R389																				W385			F391								
												G246	V393		G392																							G392								
												G247	L394		V393																							V393								
													A395																																	
VI			K79	H80	F81	I82	K83			V107	I108	S109	G110	L111	P112	A113		T115	S116	E118	F132	V134		H139	R140	L141	Y144	S146	E170	P177		N180			D184	T185	M187	F188	V189	T191	V192	V193	S195	N196	Q198	N199
PYL3-ABA-HAB1			ABA	S322	ABA	ABA	E201			ABA	G246	R199	H245	ABA	ABA	ABA		ABA	ABA	ABA	ABA	ABA		ABA	ABA	ABA	ABA	ABA	ABA	W385		I383			I383	W385	K381	ABA	ABA	F391	ABA	ABA	E323	ABA	E323	E323
4DS8.pdb				E323	T324						T324	E201	G246	Q386	W385	Q386									Q384	W385						Q384			W385		I383	I383		V393	V393		Y404			
[38]				T324	V393							E203	R389	R389	Q386										W385							W385					F391	W385		L405						
					Y404							H245	V393	V393	R389										Q386													F391								
												G246	L394	Y404	G392																							G392								
												G247	A395		V393																							V393								
VII				P57	F58		S60	R61	D78	L79	K80	S81	G82	L83	P84	A85	T86						D110		R112	L113				P144		N147			E151	T152	F154	F155		A158	L159	Q161	C162		N165	
PYL10-HAB1				G246	G246		E201	S200	S200	R199	R199	R199	H245	Q386	W385	R199	R199						Q386		Q384	W385				W385		I383			V382	W385	K381	I383		F391	V393	E323	E323		E323	
3RT0.pdb				S322	T324		S200				E201	E203	R389	R389	Q386	Q386	K491								W385							Q384			I383		I383	W385		V393			T324			
ABA-free				T324	V393						E203	D243	V393	V393	R389										Q386							W385			Q384		F391	F391					Y404			
[15]					Y404						G246	G244	L394		G392																				W385			G392								
											G247	H245	A395		V393																							V393								
												G246																																		
												D492																																		
